# Uncovering the genetic basis of antiviral polyketide limocrocin biosynthesis through heterologous expression

**DOI:** 10.1186/s12934-024-02621-9

**Published:** 2025-01-13

**Authors:** Sofiia Melnyk, Marc Stierhof, Dmytro Bratiichuk, Franziska Fries, Rolf Müller, Yuriy Rebets, Andriy Luzhetskyy, Bohdan Ostash

**Affiliations:** 1 Department of Genetics and Biotechnology, Ivan Franko National University of Lviv, Hrushevskoho st. 4, Rm. 102, Lviv, 79005 Ukraine; 2 Explogen LLC, Volodymyra Velykoho st. 16, Lviv, 79032 Ukraine; 3 Helmholtz Institute for Pharmaceutical Research Saarland (HIPS), UdS Campus, Bld. E8.1, 66123 Saarbrücken, Germany; 4 Helmholtz Institute for Pharmaceutical Research Saarland (HIPS), Helmholtz Centre for Infection Research (HZI), Saarland University, 66123 Saarbrücken, Germany; 5 German Center for Infection Research (DZIF), Partner Site Hannover-Braunschweig, 38124 Braunschweig, Germany; 6 German-Ukrainian Core of Excellence in Natural Products Research (CENtR), Zelena st. 20, Lviv, 79005 Ukraine; 7 Saarland University, HIPS UdS Campus Bldg C2.3, 66123 Saarbruecken, Germany

**Keywords:** Limocrocin, Genes, Heterologous expression, Biosynthetic gene cluster, Reverse transcriptase inhibitor

## Abstract

**Background:**

*Streptomyces roseochromogenes* NRRL 3504 produces clorobiocin, an aminocoumarin antibiotic that inhibits DNA replication. No other natural products have been isolated from this bacterium so far, despite the presence of a rich repertoire of specialized metabolite biosynthesis gene clusters (smBGCs) within its genome. Heterologous expression of smBGCs in suitable chassis speeds up the discovery of the natural products hidden behind these sets of genes.

**Results:**

In this work we focus on one intriguing smBGC of NRRL 3504 bearing some similarity to gene clusters involved in production of manumycin family polyketides. Through heterologous expression in *Streptomyces* chassis strains *S. albus* Del14 and *S. lividans* ΔYA9, this smBGC (hereafter referred to as *lim* BGC) was shown to direct the production of unusual polyketide limocrocin (LIM) known for its ability to interfere with viral reverse transcriptases. The organization of *lim* BGC, data on the structures of revealed metabolites as well as manipulations of *lim* genes allowed us to put forward an initial hypothesis about a biosynthetic pathway leading to LIM. We provide initial data on two LIM derivatives as well as updated NMR spectra for the main product.

**Conclusion:**

This study reveals the genetic control of biosynthesis of LIM that remained hidden for the last 70 years. This, in turn, opens the door to biological routes towards overproduction of LIM as well as generation of its derivatives.

**Supplementary Information:**

The online version contains supplementary material available at 10.1186/s12934-024-02621-9.

## Background

**Fig. 1:**
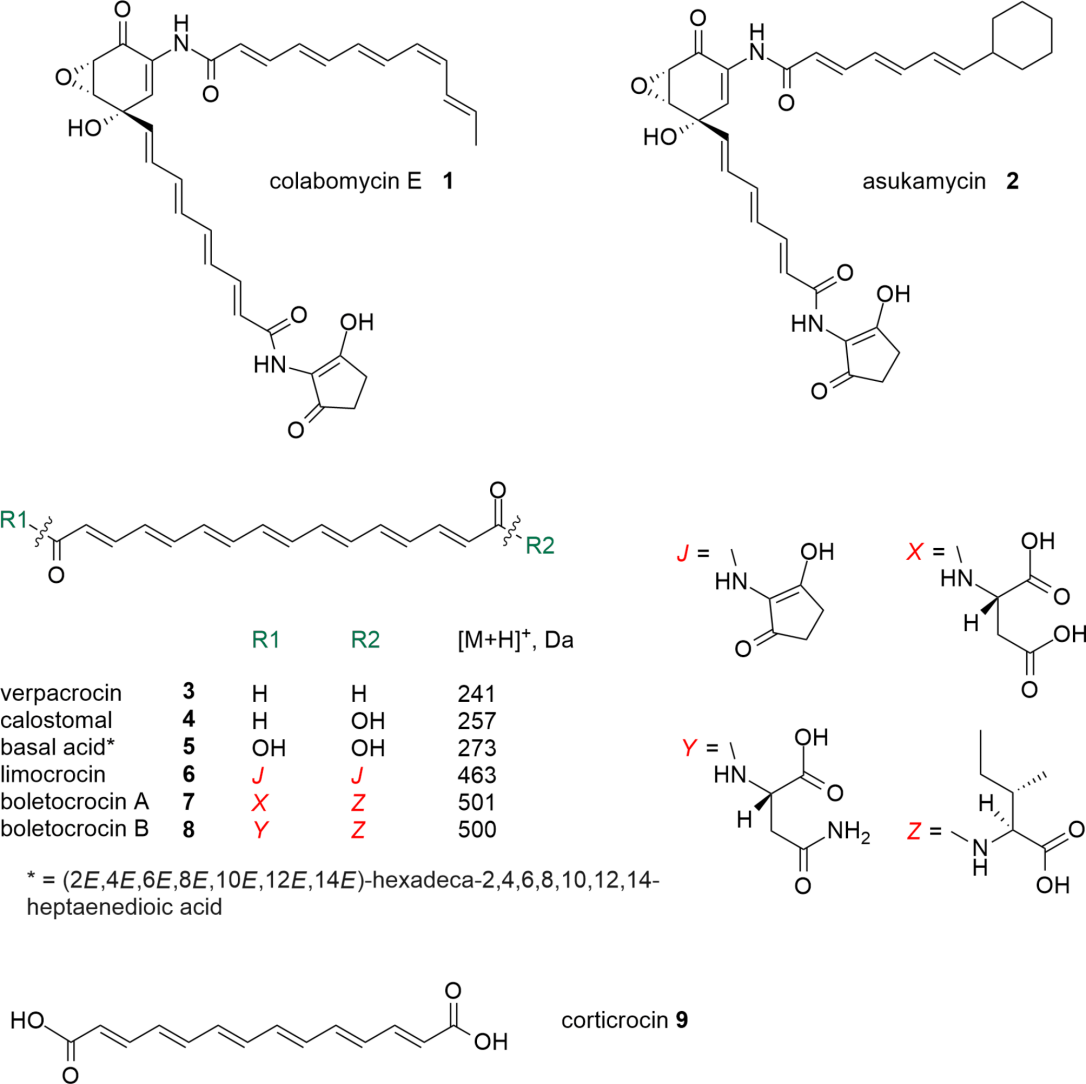
Structural formulae of compounds mentioned in the text. Compounds **3**, **4**, **7**–**9** were identified in basidiomycetes. Basal acid **5** (so far not observed in nature) can be considered a free dicarboxylic acid intermediate to the compounds **3**–**8**

*Streptomyces roseochromogenes* NRRL 3504 remains the only known producer of aminocoumarin antibiotic clorobiocin, a very potent inhibitor of DNA gyrases [1]. No other specialized metabolites are known to be produced by this strain, despite the abundance of biosynthetic gene clusters (BGCs) within its genome [2, 3]. We have become particularly interested in the chemical nature of compounds controlled by the BGC#29 [3]. The core part of the latter consists of several genes encoding unusual type III polyketide synthases (PKS) and associated proteins. The two type III PKS gene from BGC#29 are similar to recently characterized PKS AsuC3 and AsuC4 that exhibit an unprecedented iterative mode of formation of the upper polyene chain of asukamycins [4]. Asukamycins are members of the manumycin family of natural products which are produced by actinomycetes and distinguished by two carbon chains – upper and lower – that are linked in a *meta* configuration to a distinctive central epoxyquinone group [5]. Significant interest in these compounds arises from their cancerostatic and immunosuppressive effects, which result from the inhibition of various eukaryotic enzymes [6]. The antiSMASH reveals a modest degree (20%) of similarity of BGC#29 to BGCs directing the biosynthesis of manumycin family natural products, such as colabomycin E **1** and asukamycin **2** (Fig. 1). Nevertheless, the genetic architecture of BGC#29 deviates significantly from that of the manumycin BGCs [6, 7], thus suggesting that the small molecule directed by the BGC#29 differs from the aforementioned natural products. Here, using a heterologous expression approach we show that BGC#29 is responsible for the production of limocrocin (LIM) **6**, a founding member of a family of polyene dicarboxylic acids first described in 1953, whose congeners were eventually found in a number of mushrooms (see Fig. 1) [8, 9]. With the knowledge of chemical identity of the final compound encoded by BGC#29 and genetic organization of the latter, we propose a biosynthetic pathway towards LIM. Our biosynthetic hypothesis is further supported by genetic manipulations of the heterologous BGC#29-carrying hosts as well as native producer, NRRL 3504. This work unveils the genetic basis of LIM family compounds biosynthesis, opening the door for their overproduction and structural diversification.

## Results

### Identification, cloning and heterologous expression of BGC#29 from NRRL 3504

In our previous studies, a comprehensive bioinformatic analysis of *S. roseochromogenes* genome using antiSMASH and alternative tools for secondary metabolome in silico analysis (such as PRISM, DeepBGC, ARTS, SEMPI and GECCO) was conducted [3]. Our attention was drawn to region 29 (hereafter BGC#29) sharing 20% similarity to the BGC involved in production of the manumycin family polyketide colabomycin (Table S1, ESM). Colabomycin and manumycin possess two polyketide chains (see Fig. 1), and corresponding gene clusters feature polyketide synthase (PKS) genes for production of these chains as well as specific starter units [6, 7]. A cursory antiSMASH analysis of the BGC#29 showed that it harbored less PKS genes and no genes for starter units, and so could not support the assembly of two manumycin-like polyketide chains (this point will be elaborated in more details in following section of this work, on basis of verified sequence of BGC#29). We therefore decided to unveil the chemical identity of BGC#29-encoded polyketide through heterologous expression.

For this purpose, a genome library of NRRL 3504 was constructed on the basis of φC31-based integrative cosmid vector cos15AAmInt. End-sequencing of the cosmid library led to identification of cosmid 15-H11 harboring BGC#29. The cosmid was transferred by conjugation into two optimized heterologous hosts – *S. albus* Del14 and *S. lividans* ∆YA9 [10, 11]. The resulting transconjugant strains *S. albus* 15-H11 and *S. lividans* 15-H11 accumulated a bright yellow pigment, a characteristic feature for numerous polyketides with conjugated double bonds [12].

Transconjugant strains were grown on solid GYM medium and the yellow pigment was extracted with methanol. LC-MS analysis of *S. albus* 15-H11 and *S. lividans* 15-H11 extracts led to identification of three new fractions that had absorption maxima at 440 nm, *m/z* of 495, 479 and 463 [M + H]^+^, and retention time of about 8.4, 9.6 and 11.2 min, respectively (Fig. S1, ESM). Using the accurate MS data for the above compounds (Table 1) and Dictionary of Natural Products, we revealed that 462 Da compound could correspond to the polyene polyketide limocrocin (LIM); cation fragmentation pattern (Fig. S2) also agreed with such an assumption. The compounds with masses 478 and 494 Da could not be identified in the databases. The difference of 16 Da suggested that 478 Da and 494 Da compounds are likely epoxides of LIM, although their MS-MS analysis was not informative to suggest the structure. Methanol extracts from the control strains (GYM agar-grown *S. albus* Del14 and *S. lividans* ∆YA9 carrying empty cosmid vector) were colorless and did not contain any of the aforementioned mass peaks.

**Table 1:** High-resolution MS data of the identified compounds

Compound	Chemical formula	Retention time, min	m/z, [M + H]^+^		Mass error, ppm
			Calculated	Observed	
Limocrocin OX2 **11** Limocrocin OX1 **10** Limocrocin **6**	C_26_H_26_N_2_O_8_ C_26_H_26_N_2_O_7_ C_26_H_26_N_2_O_6_	8.4 9.6 11.2	495.1761 479.1812 463.1863	495.1769 479.1814 463.1868	1.61 0.41 1.07

### 462 Da compound is limocrocin

For scaled-up purification of the identified small molecules we used *S. lividans* 15-H11 because, based on the relative intensity of yellow pigmentation and MS peaks (Fig. S3), it accumulated more target compounds than *S. albus*. *S. lividans* 15-H11 was inoculated into 10 L of DNPM medium, and the culture broth was extracted with an equal amount of butanol. The compounds were purified from extract using size-exclusion and semi-preparative chromatography to achieve necessary purity for NMR structural analysis.

After a series of extraction and purification steps we were able to obtain about 0.7 mg of pure 462 Da compound. As mentioned above, searching its exact mass in the Dictionary of Natural Product database revealed LIM ([M + H]^+^ = 463.1863) as the only hit. The compound was further confirmed by UV/VIS and NMR analysis (Figs. S4-S8, ESM).

UV/Vis bands were determined during an HPLC run at λmax [nm] = 260 (shoulder), 332, and 434 nm, which is similar to the values reported in the literature (λmax (0.4 M Na_2_CO_3_) = 258 nm and 420 nm) [8].

The structure determination by NMR spectroscopy revealed that LIM is a mixture of isomers likely due to *cis*/*trans* isomerization. Due to insufficient amounts of compound, a full assignment could not be done due to the lack of adequate ^13^C-NMR and HMBC spectra. Therefore, the structural elements of LIM could only be partially assigned and confirmed by a few observable signals in the 1D and 2D experiments. LIM consists of two 2-amino-3-hydroxycyclopentenone units linked to hexadecaheptaenedioic acid via amide bonds. Based on this structure, the ratio of the combined integral values of the proton signals of methines (14xCH) to methylenes (4xCH_2_) should be 14/8 regardless of *cis/trans* isomerization. By analyzing the experimental edited-HSQC and ^1^H-NMR spectra, we were able to identify all methine and methylene signals and confirm the expected ratio of 14/8 of the integral values (Fig. S4, S7). The presence of the 2-amino-3-hydroxy-cyclopentenone moiety was confirmed by HMBC correlations observed between the methylene signals and the quaternary carbons at δ_C_ = 191–193 ppm, 171–175 ppm and 109–112 ppm (Fig. S8). A weak HMBC correlation was also observed between δ_H_ = 6.26–6.34 ppm and δ_C_ = 164 suggesting that one of the methines correlates to an amide-carbonyl group which is in accordance with the structure of LIM.

The experimental data are consistent with the literature and strongly indicate that 462 Da mass peak corresponds to LIM [13].

The other two compounds, 478 and 494 Da, could not be purified in quantities sufficient for further studies due to their instability, which we suspect is related to the highly reactive epoxide groups, rendering the compounds susceptible to degradation.

### Refining the limocrocin BGC structure

Available NRRL 3504 genome sequence, being 98.67% complete [2], harbors a number of unresolved sites – ambiguities, or gaps. Several of them are found within the BGC#29, particularly its PKS genes. The BGC borders could only be guessed from antiSMASH results. The latter is because the 40-kb fragment of NRRL 3504 genome within cosmid 15-H11 harbored mainly genes for hypothetical proteins or those falling into broad annotation categories (e.g., oxidoreductases), making it difficult to clearly determine their belonging to LIM (hereafter *lim*) BGC. We therefore performed a round of Sanger sequencing (see Methods) to obtain complete unambiguous *lim* BGC, and then looked for similar BGCs in available actinomycete genomes. Our expectation was that such BGCs will bear salient conservancy across genomic loci involved in biosynthesis of LIM congeners, and little to no similarity on the borders, thus helping better define the set of genes necessary for LIM production.

**Fig. 2:**
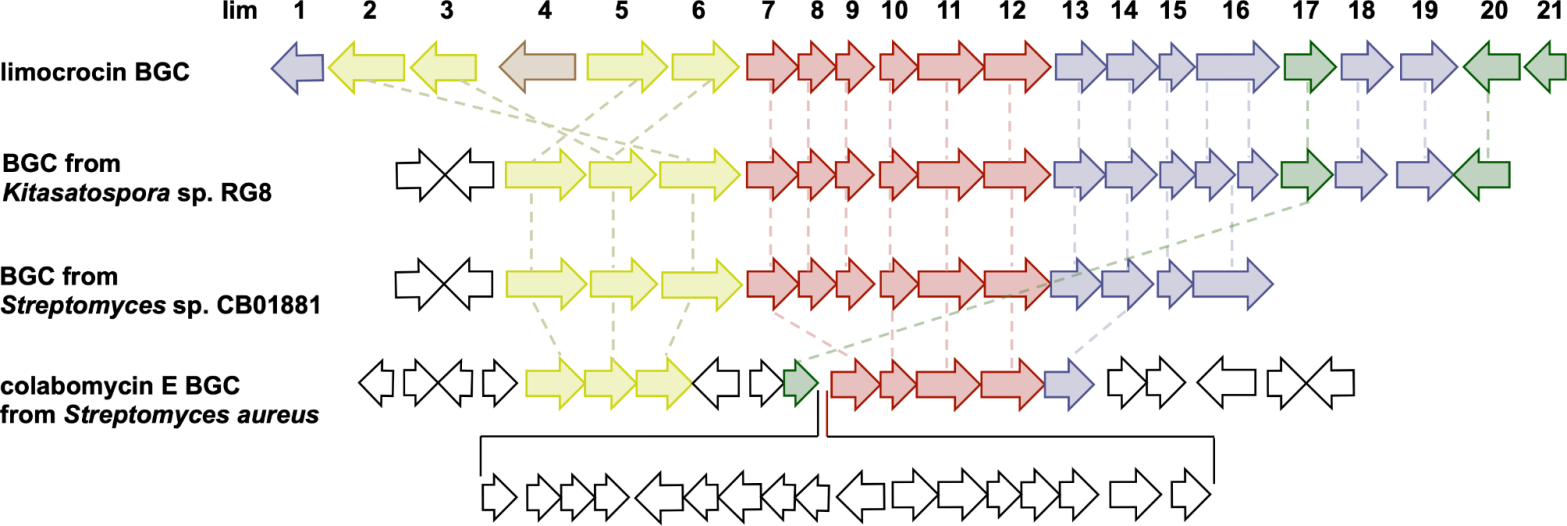
Genetic architecture of *lim* BGC and its homologs in the other actinomycetes. Color code corresponds to groups of genes involved in *lim* synthesis: C_5_N moiety (olive), PKS (burgundy), tailoring enzymes (blue), regulatory (green) and transport (brown) proteins. Genes not homologous or similar to known precedents are shown as white arrows. Homologous genes are connected by dashed lines

Indeed, ClusterBlast [14] revealed BGCs highly syntenous to *lim* gene cluster within genomes of *Kitasatospora* sp. RG8 and *Streptomyces* sp. CB01881, leading to clearer delineation of the extent of *lim* BGC (Fig. 2; ESM Table S1). Thus, *lim* BGC contains a set of genes responsible for the synthesis of the polyketide chain (*lim7-lim12*), several tailoring genes for chain modification (*lim1*, *lim13-liml6*, *lim18-lim19*), genes responsible for C_5_N moiety synthesis (*lim2/lim3*,* lim5/lim6*), three genes coding regulators (*lim17*,* lim20 and lim21*) and a transporter gene *lim4*. One outstanding feature of the *S. roseochromogenes* NRRL3504 *lim* BGC is that it harbors two copies of 5-aminolevulinate synthase (5-ALAS) gene, *lim3* and *lim6*, likely a result of rearrangements with the cluster, which also led to concomitant relocation of long-chain fatty acid-CoA ligase gene (*lim2*). Proposed functions of all *lim* genes are listed in Table 2.

### NRRL3504 produces limocrocin

Having revealed that BGC#29 directs the production of LIM, we wondered whether LIM can be detected in *S. roseochromogenes* NRRL 3504. We checked a large collection of LC-MS data from our previous studies of clorobiocin biosynthesis (https://data.mendeley.com/datasets/xwfmffkmg5/1*)*, but no LIM-associated mass-peaks were revealed. This could be due to the fact that past cultivation conditions did not support LIM production, and we used ethyl acetate instead of methanol to extract specialized metabolites. Therefore, the conditions developed for heterologous hosts (e.g. solid GYM for growth and methanol for extraction) were recapitulated in NRRL 3504. Indeed, we were able to obtain a yellow-colored compound from NRRL 3504, which yielded a fraction with retention time and exact mass identical to LIM. The 478 and 494 Da derivatives were also present (Fig. S9). The NRRL3504 extracts did not contain putative LIM intermediates, such as acid **5**, as well as the one carrying a single C_5_N unit.

**Table 2:** Predicted functions of open reading frames of *lim* BGC

Protein	AA	Accession	Homologue	%ID	Predicted function
Lim1	286	WP_063750623.1	PntD (ADO85576.1)	27	TauD/TfdA family dioxygenase
Lim2	530	WP_245238157.1	ColD3 (AIL50189.1)	58	long-chain fatty acid-CoA ligase
Lim3	409	WP_023548480.1	ColD2 (AIL50190.1)	67	5-aminolevulinate synthase
Lim4	518	WP_051430097.1	Mem2 (CAM56779.1)	43	MFS transporter
Lim5	546	WP_280923667.1	ColD1 (AIL50191.1)	54	AMP-binding protein
Lim6	412	WP_023548486.1	ColD2 (AIL50190.1)	73	5-aminolevulinate synthase
Lim7	246	WP_031225354.1	ColC10 (AIL50168.1)	56	3-oxoacyl-[acyl-carrier-protein] reductase
Lim8	137	WP_023548490.1	SapF (QMX85613.1)	43	beta-hydroxyacyl-ACP dehydratase
Lim9	158	WP_023548491.1	SapG (QMX85614.1)	57	beta-hydroxyacyl-ACP dehydratase
Lim10	86	WP_023548493.1	ColC5 (AIL50167.1)	61	acyl carrier protein
Lim11	403	WP_023548499.1	ColC4 (AIL50166.1)	55	beta-ketoacyl-[acyl-carrier-protein] synthase
Lim12	373	WP_245238402.1	ColC3 (AIL50165.1)	50	beta-ketoacyl synthase
Lim13	254	WP_051430098.1	SapL (QMX85619.1)	49	alpha/beta hydrolase
Lim14	212	WP_023548505.1	ColC16 (AIL50164.1)	53	DsbA family protein
Lim15	57	WP_023548508.1	ColC16 (AIL50164.1)	56	DsbA family protein
Lim16	477	WP_023548510.1	MmcL (AAD32735.1)	41	aldehyde dehydrogenase
Lim17	217	WP_023548512.1	ColR1 (AIL50186.1)	52	response regulator transcription factor
Lim18	176	WP_023548514.1	Fma-ABM (EAL85120.1)	33	ABM family monooxygenase
Lim19	272	WP_023548516.1			thioesterase
Lim20	256	WP_023548518.1	Cip3 (QNH67531.1)	42	response regulator transcription factor
Lim21	122	WP_280923686.1	CtcS (AEI98662.1)	32	MarR family transcriptional regulator

### Genetic manipulations of LIM BGC in heterologous and native hosts

**Fig. 3:**
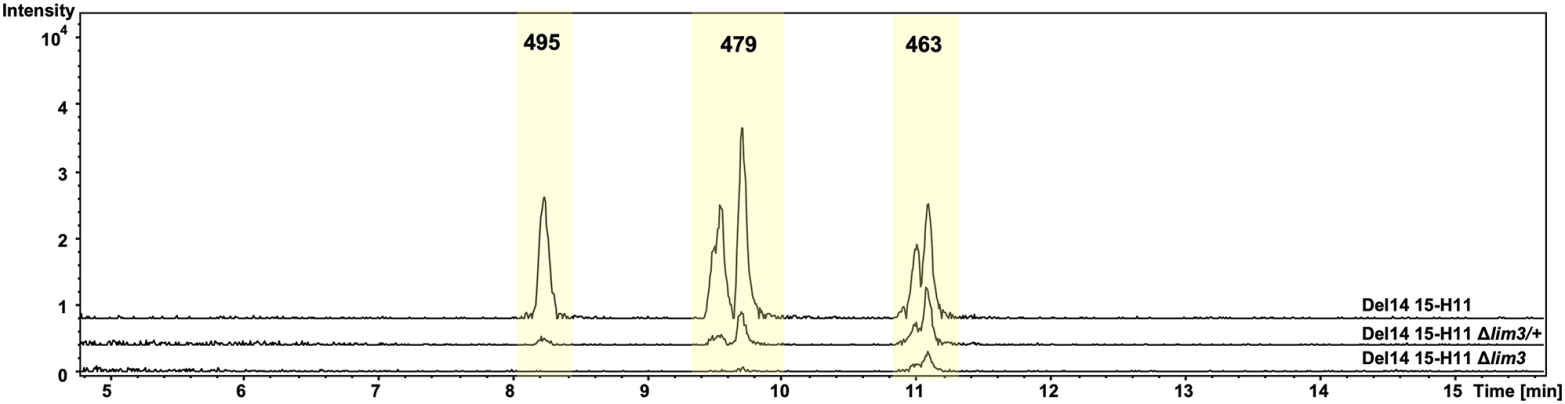
The *lim3* deletion affects biosynthesis of LIM 463 *m/z* and its oxidized derivatives 479 *m/z* and 495 *m/z* ([M + H]^+^). LC-MS traces of Del14 strains harboring intact cosmid with *lim* BGC (Del14 15-H11), deletion of *lim3* (Del14 15-H11 Δ*lim3*) and complementation of *lim3* deletion with *lim3* on plasmid (Del14 15-H11 Δ*lim3*/+). The *y* axis, relative abundance, arbitrary units. Trace chromatograms represent typical result of three independent experiments

We generated a derivative of cosmid 15-H11, 15-H11∆lim3, where the *lim3* gene for 5-aminolevulinate synthase was replaced with a hygromycin resistance cassette. Comparative LC-MS analysis revealed that 15-H11∆lim3-carrying Del14 still produced LIM, at about 20% of the level of the strain expressing parental cosmid; production of 478 and 494 Da LIM derivatives were extremely reduced (to 3% and 1%, respectively). Co-expression of 15-H11∆lim3 and *lim3* partially restored the production of LIM and its oxidized derivatives, albeit clearly not to the initial level (Fig. 3). Given the genetic organization of *lim* gene cluster (see Fig. 2), it is likely that *lim3* replacement exerted a polar effect on the expression of downstream *lim* genes. Particularly, perturbed expression of *lim2* would reduce the level of production of C_5_N unit, and so overall yield of limocrocins. An almost complete cessation of production of oxidized LIM derivatives by the 15-H11∆lim3 and 15-H11∆lim3/+ (*lim3*-expressing) strains suggests tentatively that the *lim1* gene might control the conversion of LIM into 478 and 494 Da compounds.

We also generated a cosmid 15-H11∆lim6, where the *lim6* gene, an apparent *lim3* paralog, was replaced with a hygromycin resistance cassette. Introduction of 15-H11∆lim6 into *S. albus* Del14 or *S. lividans* ∆YA9 failed despite numerous attempts and variations in conjugation protocol. Although small transconjugant colonies were visible after 3 days of incubation, they did not reach sporulation stage and did not grow after re-streaking onto fresh plates (likely due to viability issues, please see the Discussion).

**Fig. 4:**
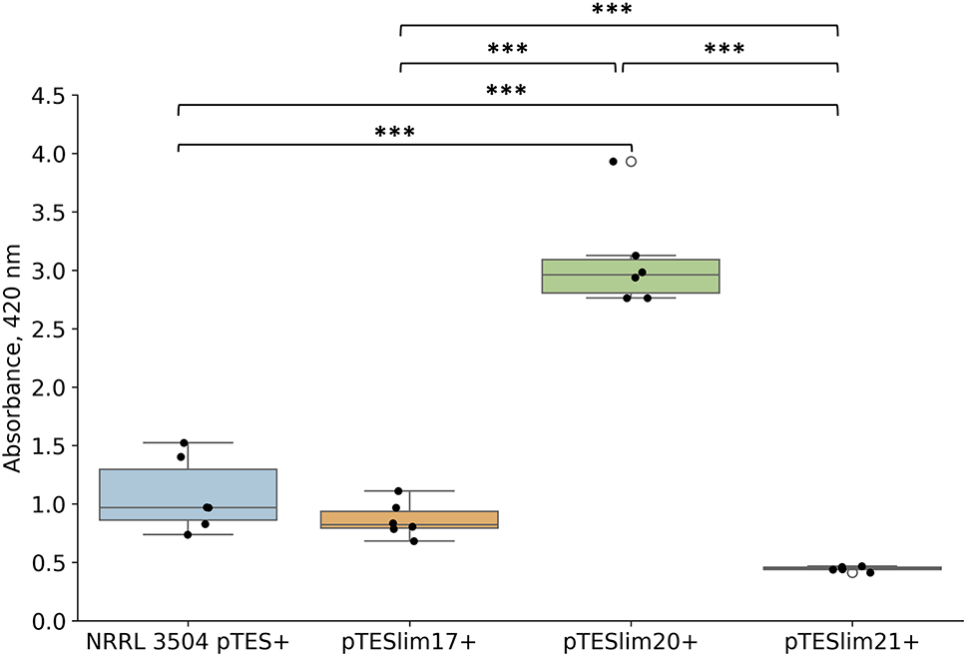
Level of LIM production by NRRL 3504 expressing *lim* cluster-situated regulatory genes *lim17*, *lim20* and *lim21*; NRRL 3504 carrying pTES vector was used as a control. A boxplot chart illustrates the absorbance values of samples at 420 nm; individual measurements are shown as black dots. Error bars, ±2SD; ***p < 0,001 (ANOVA)

On the basis of integrative vector pTES (see Methods) we constructed three plasmids for ermEp*-driven constitutive expression of *lim* cluster-situated regulatory genes. These included *lim20* for response regulator transcription factor Lim20 of LuxR family [15] (plasmid pTESlim20); *lim17* (pTESlim17), a homolog of the *colR1* gene encoding the principal regulator of the colabomycin pathway [5]; and *lim21* for MarR family transcriptional regulator (pTESlim21). The levels of LIM production by NRRL3504 strains carrying empty vector pTES and each of the aforementioned plasmids were compared. The results are presented in Fig. 4. Of the three tested genes only *lim20* had a significant positive effect on LIM titers; the pTESlim20^+^ strain produced LIM roughly two times more than the control strain. The level of LIM synthesis in NRRL 3504 carrying pTESlim21 was reproducibly lower than in the control strain, while LIM titers for pTESlim17^+^ strain were within the error margin of production level of the control.

### Proposed LIM biosynthetic pathway

**Fig. 5:**
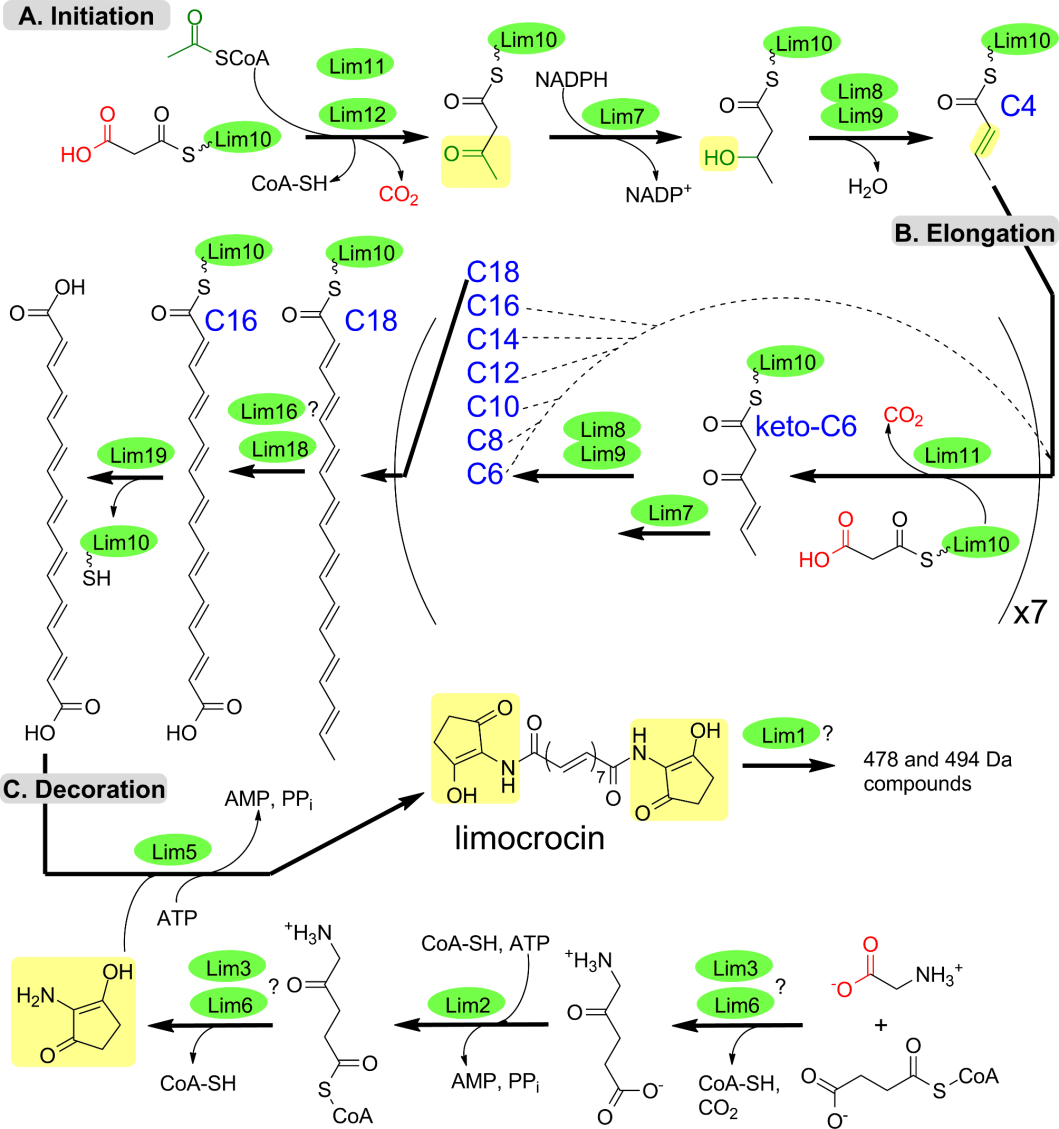
Proposed pathway for LIM biosynthesis. Solid arrows indicate the order of LIM assembly. Dashed lines mark the iterative process of conversion of C12 chain into C18 one, through a series of two-carbon intermediates. LIM biosynthetic enzymes are shown as green ovals. Carboxylic groups that leave the pathway as CO_2_ are shown in red. Pale yellow background indicates positions of new moieties in the intermediates. Interrogation sign marks enzymes which could be dispensable for LIM production (see the Discussion)

A hypothetical pathway towards LIM is shown in Fig. 5. It takes into account the data on in vitro reconstruction of initial steps of asukamycin biosynthesis [4, 7], our LC-MS results for the heterologous and native LIM producers, as well as genetic and bioinformatic scrutiny of the *lim* genes.

**Fig. 6:**
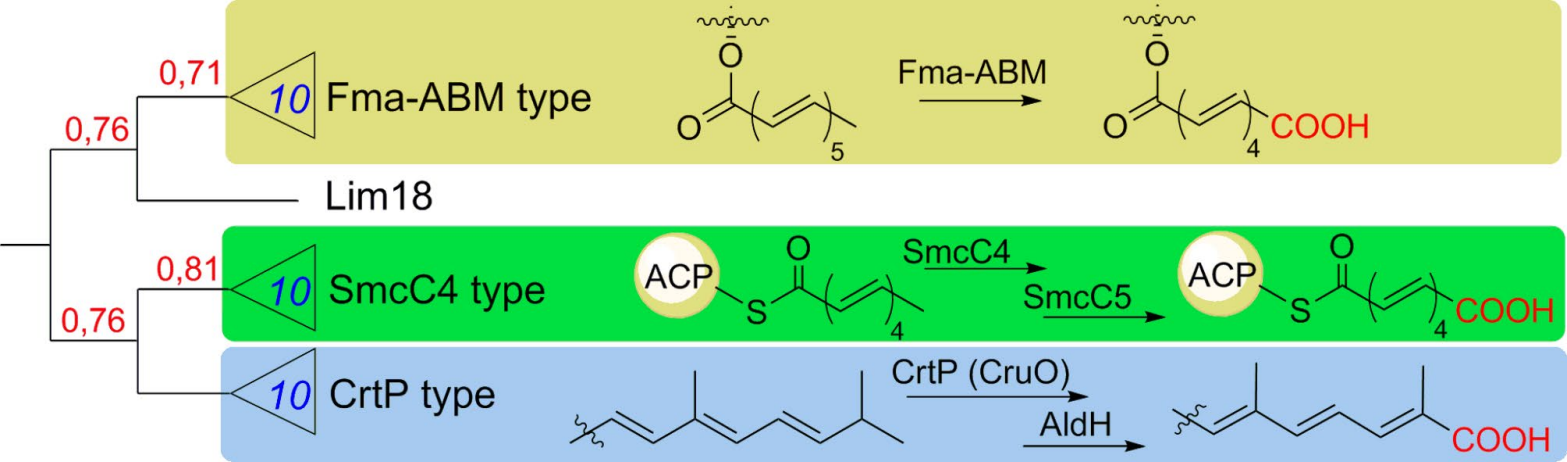
Maximum likelihood tree of Lim18 protein and its functional counterparts from various specialized metabolic pathways. Numbers on the nodes (in red) are bootstrap values of tree topology reliability (with 1,0 being maximal value; see Methods). Blue numbers on the collapsed clades show the number of proteins within the clade. See Fig. S10 (ESM) for full (uncollapsed) tree. Colored background marks three distinct clades, proteins that exemplify the clade and its verified (Fma-ABM, CruO) or proposed (SmcC4) reaction mechanism (see the Discussion). SmcC4 and CrtP fall into family of carotenoid oxygenases, Fma-ABM functions as monooxygenase

Below we briefly enumerate major points of our biosynthetic hypothesis. An overall mechanistic picture of LIM dicarboxylic acid production resembles that recently described for the upper chain of asukamycin [4]. It involves ACP Lim10, ketosynthases Lim11 and Lim12, and tailoring enzymes Lim7-9. The distinct features of our proposal are in the initiation stage and the final steps leading to release of the full polyketide chain from ACP. The assembly of LIM polyketide moiety is likely initiated with one acetyl-CoA unit and one malonyl-ACP, as it offers the most parsimonious route towards even-chained (C18) polyene. The *lim* BGC encodes monooxygenase Lim18 whose homolog Fma-ABM (Fig. 6; Fig. S10) in fumagillin pathway catalyzes oxidative cleavage of unsaturated twelve-carbon chain, leading to formation of decatetraenedioic ester [16]. By analogy we propose that Lim18 (alone or in combination with aldehyde dehydrogenase/reductase Lim16) cleaves C18 intermediate (see Fig. 5) to give C16 dicarboxylic intermediate tethered to ACP Lim10. The *lim* BGC also harbors gene *lim19* for thioesterase likely involved in the release of dicarboxylic acid intermediate **5** (Fig. 1). Subsequent decoration of acid **5** with C_5_N unit follows the established biochemical logic [17]. Lim1 is likely responsible for the formation of oxidized 478 and 494 Da derivatives of LIM, although their exact chemical structure remains to be elucidated.

### Biological activity of LIM congeners

We determined the biological activities of LIM and its oxidized 478 Da derivative against a number of bacterial and fungal strains (Table 3A). No activity was detected against Gram-positive (*B. subtilis*, *S. aureus*) and Gram-negative indicator strains (*E. coli*) as well as against the fungi *Mucor hiemalis* (in all cases MIC > 64 µg/mL). These results are in agreement with literature data where LIM showed only weak activity against *Bacillus anthracis* [18]. In addition, we revealed no cytotoxicity for LIM and only slight toxicity for 478 Da compound toward chinese hamster ovary cells (Table 3B; Fig. S11).

**Table 3:** Testing of biological activity and cytotoxicity of limocrocins. **A** antimicrobial activities of limocrocins against selected microorganisms. **B** half maximal inhibitory concentrations (IC_50_) ± standard deviation against Chinese hamster ovary cells (CHO-K1)

**A**	MIC [µg/mL]	
	Limocrocin 6 (462)	Limocrocin OX1 10 (478)
*B. subtilis* DSM10	> 64	> 64
*S. aureus* ATCC29213	> 64	> 64
*E. coli* BW25113 (WT)	> 64	> 64
*E. coli* JW0451-2 ΔacrB*	> 64	> 64
*M. hiemalis* DSM2656	> 64	> 64
*efflux-deficient		
**B**	IC_50_ [µg/mL]	
Limocrocin **6** (462)	> 37	
Limocrocin OX1 **10** (478)	30.3 ± 4.5	
Doxorubicin	0.067 ± 0.026	

## Discussion

Actinomycete genomes harbor dozens of BGCs for the production of diverse specialized metabolites. Majority of these BGCs, however, remain cryptic under typical laboratory conditions. Heterologous expression of genomic or metagenomic DNA in well-characterized and easy-to-use microorganisms (known as expression chassis strains) proved itself as a powerful approach that facilitates the identification, purification and bioactivity investigation of new small molecules [19]. Here, taking this approach, we discovered and studied the gene cluster for production of LIM. This natural product was first described in the 1950s as a yellow pigment accumulated by *Streptomyces limosus* and later, in the 1980s, was shown to exhibit activity against the reverse transcriptase of avian myeloblastosis virus [8, 18]. LIM is an archetypal member of the family of unusual polyene dicarboxylic acids that undergo various modifications of carboxyl groups. Rare fatty acids are an underexplored niche in natural product research despite a wide range of biological activities [9]. Unusual (2*E*,4*E*,6*E*,8*E*,10*E*,12*E*,14*E*)-hexadeca-2,4,6,8,10,12,14-heptaenedioic acid (**5**) is a core part of LIM as well as a number of LIM congeners from fungi, such as verpacrocin (**3**) from ascomycete *Verpa digitaliformis*, boletocrocin A (**7)** and B (**8**) from the Japanese mushroom *Boletus laetissimus* and calostomal (**4**) from the gasteromycete *Calostoma cinnabarinum* [20–22], and closely related corticrocin **9** isolated from the yellow mycorrhiza fungus *Corticium croceum* [23]. The lack of genomic data of these fungal species makes it impossible to look for homologs of LIM biosynthetic enzymes. BLASTP-assisted search against all available basidiomycete genomes, using different LIM biosynthetic proteins as a query, did not return similar sequences. Besides the lack of appropriate genomic data, failure to reveal homologous sequences in mushrooms could indicate that biosynthesis of compounds **3**, **4**, **7**–**9** follows a logic different from the one exemplified by *lim* BGC. Precedents of operation of different biosynthetic routes in different species towards identical natural products are well known in fungi and bacteria [24]. Thus, at the moment, our work sheds the light on the biosynthesis of LIM family compounds in bacteria, which had remained unknown for seven decades, and offers a convenient biological platform to overproduce LIM and generate its analogs. It remains to be studied to what extent the biosynthesis of fungal LIM congeners resembles the biochemical logic of LIM production by bacteria.

Besides LIM, the extracts of both native and heterologous host contain fractions for 478 and 494 Da compounds. Сonjugated double bonds of the polyene chain are highly reactive which leads to the formation of a variety of oxidized products [25]. The exact mass of the aforementioned compounds is consistent with LIM derivatives carrying one (478) and two (494) epoxide moieties. Under collision-induced dissociation conditions used in our experiments the 478 and 494 Da compounds fragmented poorly, precluding any structure inference. Identifying the structure of oxidized derivatives could be challenging due to the complexity and chemical instability of oxidation products, the formation of isomers, analytical limitations and sample impurities.

A peculiar feature of *lim* BGC is the presence of two 5-aminolevulinate synthase (5-ALAS) genes, *lim3* and *lim6*, presumably involved in the initial steps of formation of 2-amino-3-hydroxycyclopent-2-enone (C_5_N) ring (see Fig. 4). The *lim3* deletion caused reduced LIM titer to 20% of the initial level, likely due to presence of paralogous *lim6* gene. Accumulation of 478 and 494 Da compounds dropped to the detection limit. We speculate that replacement of *lim3* with hygromycin resistance gene exerts a polar effect on the expression of downstream *lim2* and *lim1* genes, likely sharing a promoter with *lim3* (see Fig. 2). In this scenario impaired *lim2* expression would lower the availability of C_5_N unit, effectively bottlenecking LIM production regardless of the level of *lim3*/*lim6* expression. Then the decreased expression of *lim1* gene for putative dioxygenase gene would lower the titer of 478 Da and 494 Da derivatives of LIM. The complementation of *lim3* mutant with the intact *lim3* gene restored the initial LIM production level only partially. Although this result may be construed as a support for polar effect hypothesis, at the moment we cannot completely rule out experimental caveats (choice of vector, inducer concentration etc.) associated with this complementation. An unexpected complexity of the role of ALAS genes in *lim* BGC is further underscored by the failure to generate host strains carrying the cosmid with *lim6* deletion. One possible explanation is that the *lim6* and *lim3* have distinct roles in LIM biosynthesis; endogenous production of either **5**, or as-yet-unknown intermediate resulting from *lim6* knockout, might be toxic to *Streptomyces*. A number of unsaturated fatty acids, such as oleic and linoleic acids, have shown a high reduction in plasmid transfer and conjugation inhibition in bacteria [26, 27]. In this regard we note that to date **5** was not recovered from natural sources, although its shorter analog, corticrocin **9**, was isolated from fungi [23].

The organization of *lim* BGC, data on the structures of revealed metabolites as well as results of gene knockouts allowed us to propose an initial hypothesis about a biosynthetic pathway leading to LIM. The production mechanism of LIM dicarboxylic acid closely resembles the process recently described for the upper chain of asukamycin. The topics of interest in case of the LIM polyketide biosynthesis are the nature of the starter unit and a formation of dicarboxylic structure, e.g. anticipated intermediate **5**. Acetyl-CoA as the starter unit offers the straightforward route towards the C18 polyene chain. The *lim* BGC encodes monooxygenase Lim18 similar to oxygenase Fma-ABM from fumagillin biosynthetic pathway. Fma-ABM represents a new class of oxygenases catalyzing both cleavage of the carbon chain and further oxidation of aldehyde intermediate to ester [16]. Following the Fma-ABM reaction mechanism, Lim18 might single-handedly cleave and oxidize the C18 chain, yielding C16 dicarboxylic intermediate tethered to ACP Lim10. In the context of the aforementioned Fma-ABM mechanism the role of putative aldehyde dehydrogenase Lim16 in LIM biosynthesis remains unknown. An alternative possibility is that Lim16 partners with Lim18 in the process of oxidative cleavage of the polyene chain, as it was proposed for decatetraene dicarboxylic chain formation in a pathway towards simocyclinone [28]. In that scenario Lim18, although bearing more sequence similarity to Fma-ABM than to SimC4/SmcC4 (see Fig. 6), still functions like the latter enzyme. Namely, Lim18 would catalyze the formation of C16 aldehyde intermediate, which is then converted to **5** through action of Lim16. Consequently, accumulation of aldehyde-featuring calostomal **4** by some fungi can be interpreted as either a loss or absence of Lim16 function in the respective producers. The described above mechanism raises the question about Lim18 mechanism, e.g. whether it either cleaves and oxidizes the polyene chain, or just oxidizes the terminal carbon, as proposed for SmcC4 [28]. It cannot be ruled out at the moment that terminal oxidation occurs after the release of polyketide chain from ACP. The genetic and biochemical characterization of Lim18 and Lim16 is therefore of special interest. It will shed light not only on the order of oxidation and release steps in the final stage of LIM assembly, but also on details of polyketide chain synthesis. Indeed, alternative starter units other than acetyl-CoA cannot be excluded, given that oxidase Lim18 may cleave off either odd or longer than two-carbon moieties. On the other hand, if Lim18 does not in fact carry out carbon chain cleavage, then there is no need for the longer chain (C18) precursor, as it is proposed in our current biosynthetic hypothesis (see Fig. 4). Furthermore, a few more genes within *lim* gene cluster have no assigned function in LIM biosynthesis. These include genes for alpha/beta hydrolase Lim13 and DsbA family proteins Lim14/15. Due to its hydrolytic activity Lim13 can act to release, modify, or structurally tailor the compound. Lim14 and Lim15 could be important for facilitating isomerization and stabilizing LIM intermediates, contributing to the specificity and efficiency of the biosynthetic pathway. We also note that current delineation of *lim* BGC, as depicted in Fig. 2, does not exclude the presence of additional regulatory or structural genes in the flanking regions. Availability of a convenient heterologous platform for LIM biosynthesis creates a fertile ground to study all the intriguing aspects of the genetics and biochemistry of this group of small molecules.

## Conclusion

Through heterologous expression we identified, for the first time, the BGC for an unusual linear polyene, limocrocin (LIM), whose biosynthesis remained hidden for seven decades until this report. Two new LIM derivatives, likely a result of polyene chain oxidation, were discovered. We further show that under typical laboratory conditions the native carrier of LIM BGC, *S. roseochromogenes* NRRL 3504, also accumulates limocrocins. On combining the results of in silico analysis of *lim* genes, their manipulations in streptomycetes and literature data, we put forward an initial hypothesis about a biosynthetic pathway leading to LIM.

## Materials and methods

### Bacterial strains and growth media

All strains and plasmids used in this work are listed in Table 4. *S. roseochromogenes* NRRL 3504 was used as a source of DNA for cosmid library construction. *S. albus* Del14 and *S. lividans* ∆YA9 were used as host strains for heterologous expression. *E. coli* EPI300-T1^R^ cells were used for preparation of NRRL 3504 cosmid library. *E. coli* WM6026 was used as a donor strain for intergeneric conjugations. *Streptomyces* strains were routinely maintained on SFM (mannitol soy flour) agar [29] at 30 °C, while GYM (glucose − 4 g, yeast extract − 4 g, malt extract − 10 g, CaCO_3_ − 2 g, agar − 12 g, distilled water − 1 L; pH 7.2) was used for heterologous production of LIM. Liquid DNPM [30] was used for LIM large-scale production via submerged fermentation.

**Table 4:** Plasmids and bacterial strains used in this work

Name	Characteristic	Reference
Plasmids		
cos15AAmInt	Am^r^; φC31-based integrative vector; p15A ori	Prof. A. Luzhetskyy
15-H11 pHET152	Am^r^; *lim* BGC cosmid Hyg^R^; pSET152 with *aac(3)IV* substituted with *hyg*	This work [34]
15-H11∆lim3	Am^r^, Hyg^r^; *lim* BGC cosmid with replacement of *lim3* gene by hygromycin cassette	This work
pIJ6902	Am^r^, Tsr^r^; φC31-based *Streptomyces* integrative vector; expression of cloned gene from thiostrepton-inducible promoter *tipAp*	[35]
pIJ6902lim3	Am^r^, Tsr^r^; *lim3* cloned into *Xba*I/*Eco*RI-cleaved pIJ6902	This work
pTES	Am^r^; φC31-based *Streptomyces* integrative vector; expression of cloned gene from *ermEp*	[36]
pTESlim17	Am^r^; *lim17* cloned into *Eco*RV/*Eco*RI-digested pTES	This work
pTESlim20	Am^r^; *lim20* cloned into *Kpn*I/*Bgl*II-digested pTES	
pTESlim21	Am^r^; *lim21* cloned into *Eco*RV/*Eco*RI-digested pTES	This work
**Bacterial strains**		
*E. coli* GB2005	DH10B derived strain with deletion of *fhuA*, *ybcC* and *recET*; a strain for routine cloning	[37]
*E. coli* WM6026	Auxotroph for 2,6-diaminopimelic acid (DAP); *lacI*^q^* rrnB3* Δ*lacZ*4787 *hsdR514 ΔaraBAD567 ΔrhaBAD568 rph-1 att*λ::pAE12 (Δ*ori*R6K-*cat*::Frt5) Δ*endA*::Frt *uidA*(ΔMluI)::*pir att*HK::pJK1006Δ(*ori*R6K-*cat*::Frt5 *trfA*::Frt) a donor strain for conjugative transfer of cosmid DNA	[38]
*E. coli* EPI300™-T1^R^	F^−^ *mcrA* Δ(*mrr-hsd*RMS-*mcr*BC) (Str^R^) Ф80d*lac*ZΔM15 Δlac*X74 rec*A1 *end*A1 *ara*D139 Δ(*ara, leu*)7697 *gal*U *gal*K λ^−^ *rpsL nupG trfA tonA dhfr* a strain for cosmid library preparation	LGC Biosearch Technologies, UK
*S. roseochromogenes* NRRL 3504	Source of DNA for cosmid library construction	ARS collection (NRRL)
*S. albus* Del14	*S. albus* J1074 with deletion of 15 BGCs; host strain for heterologous expression	[10]
*S. lividans* ∆YA9	*S. lividans* TK24 with deletion of 9 BGCs; host strain for heterologous expression	[11]
*S. albus* Del14 15-H11	Chassis strain carrying *lim* BGC cosmid	This work
*S. lividans* ∆YA9 15-H11	Chassis strain carrying *lim* BGC cosmid	This work
*S. albus* Del14 15-H11 *∆lim3*	Chassis strain carrying *lim* BGC cosmid with deletion of *lim3*	This work
*S. albus* Del14 15-H11 ∆*lim3*/+	*S. albus* Del14 15-H11 ∆*lim3* with complementation of *lim3* deletion	This work
*S. roseochromogenes* pTESlim17	NRRL 3504 derivative carrying pTESlim17	This work
*S. roseochromogenes* pTESlim20	NRRL 3504 derivative carrying pTESlim20	This work
*S. roseochromogenes* pTESlim21	NRRL 3504 derivative carrying pTESlim21	This work

For genomic DNA isolation *S. roseochromogenes* strain was cultivated in liquid TSB (tryptic soy broth) medium (Condalab, Madrid, Spain) for 48 h. Recombinant *Streptomyces* strains were cultivated in presence of 50 µg/mL of apramycin sulfate and 100 µg/mL of hygromycin when appropriate. *E. coli* strains were grown at 37 °C in liquid or agar LB (lysogeny broth) medium [31], supplemented with 50 µg/mL of apramycin sulfate, 100 µg/mL of hygromycin, 50 µg/mL of thiostrepton and 19 µg/mL 2,6-diaminopimelic acid when appropriate.

### Construction and screening of cosmid library

Genomic DNA of *S. roseochromogenes* was isolated using phenol-chloroform method [32]. DNA fragments of approximate size of 35–40 kb were ligated into *PmeI* site of vector cos15AAmInt containing ϕC31 integrase cassette. The ligated DNA was packaged into phage particles and then used to infect *E. coli* EPI300 cells (TransforMax™ EPI300™-T1^R^ Electrocompetent *E. coli* Kit, Biosearch Technologies). The cosmid library of 1600 colonies was end-sequenced. Cosmid carrying manumycin-like BGC (15-H11) was checked by *Bam*HI restriction and sequenced using primers cos15AChF and cos15AChR. According to sequencing results, 15-H11 includes 40 kb of genomic DNA (coordinates from 4,684,247 to 4,724,436 on *S. roseochromogenes* chromosome). The *lim* BGC is located in the central region of the cosmid insert and flanked by approximately 10 kb of the NRRL 3504 chromosome on either side. Complete ungapped sequence of *lim* BGC was deposited into NCBI under accession number BK068727.

### Expression of BGC#29 in heterologous hosts

A standard intergeneric mating protocol [29] was used to transfer cosmid 15-H11 from *E. coli* WM6026 into heterologous hosts – *S. albus* Del14 and *S. lividans* ∆YA9. Briefly, pre-germinated at 50 °C for 10 min *Streptomyces* spores were cooled down to room temperature and mixed with cells of an overnight culture of donor *E. coli* WM6026 harboring 15-H11. Mixed cell suspensions were plated on well-dried SFM plates supplemented with 10 mM MgCl_2_. After 8 h of incubation at 30 °C, plates were overlaid with 1 mL of sterile distilled water containing 50 µg/mL of apramycin sulfate and 50 µg/mL fosfomycin. Antibiotic-resistant transconjugants were analyzed after 120 h of growth.

### Generation of recombinant plasmids

The oligonucleotides used for generating the recombinant plasmids are listed in ESM Table S2. Recombinant plasmids for *lim17*,* lim20* and *lim21* expression were constructed using the same approach. Coding sequences of *lim17* (651 bp), *lim20* (768 bp) and *lim21* (366 bp) were amplified from *lim* cosmid 15-H11 using lim17_EcoRV_F/lim17_EcoRI_R, lim20_KpnI_F/lim20_BglII_R and lim21_EcoRV_F/lim21_EcoRI_R pairs of primers, respectively. The inserts *lim17* and *lim21* were digested with *Eco*RV and *Eco*RI restriction endonucleases, *lim20* – with *Kpn*I and *Bgl*II, and cloned into pTES vector (under the control of *ermEp*) via *Eco*RV/*Eco*RI and *Kpn*I/*Bgl*II sites, respectively. As a result, we obtained plasmids pTESlim17, pTESlim20 and pTESlim21, carrying the corresponding genes.

Gene deletion was carried out via the Red/ET recombination approach [33] using cosmid 15-H11 and hygromycin resistance cassette from plasmid pHET152. Hygromycin cassette was amplified from pHET152 using Δlim3hyg_F/Δlim3hyg_R and Δlim6hyg_F/Δlim6hyg_R pairs of primers. Gene deletions were confirmed by PCR using Δlim3_ChF/Δlim3_ChR and Δlim6_ChF/Δlim6_ChR pairs of primers. For complementation of deletion, coding sequence of *lim3* (1,227 bp) was amplified from 15-H11 using lim3_XbaI_F/lim3_EcoRI_R primers, digested with *Xba*I and *Eco*RI restriction endonucleases and cloned into φC31-based pIJ6902 vector [29] under control of thiostrepton-inducible promoter *tipAp* to give pIJ6902lim3. As the main *attB*^φC31^sites in Del14 are occupied with cosmid 15-H11∆lim3, pIJ6902lim3 will detour to additional pseudo-*attB* sites known to be present in J1074 genome [39]. This vector allows the use of thiostrepton for selection (in the background of Am^r^ and Hyg^r^ phenotypes of the chassis strain) and inducible expression purposes. As to the latter, we tested 5 (usual concentration for thiostrepton as an inducer [29]), 25 and 50 µg/mL of the antibiotic, which did not differ in terms of LIM accumulation. The results reported in this work were obtained at the highest aforementioned concentration.

### Extraction of limocrocins from agar-grown cultures

For metabolite extraction *S. albus* 15-H11 and *S. lividans* 15-H11 were incubated for 6 days at 30 °C on GYM agar. Half of the dish was chopped into 1 cm^2^ pieces and extracted with 8 ml of methanol. Mixtures were left for 2 h at the orbital shaker (220 rpm) at room temperature, and then fractionated by centrifuging 10 min at 6,000 rpm. The bright-yellow organic (upper) phase was collected and, finally, 2 mL of the organic phase were evaporated *in vacuo*.

### Absorbance spectroscopy of extracts

Absorbance spectra of LIM extracts were recorded using a VWR^®^ M4, UV/Visible Spectrophotometer. Methanol extracts were generated as described above, from equal amounts of the NRRL 3504 biomass. The absorbance (A) was measured over the wavelength range of 400 to 440 nm with measurements taken at 1 nm increments. The A_420_ data were exported and analyzed using Microsoft Excel. The data represent mean values of six independent biological experiments. The spectroscopy data agree with the LC-MS analysis of the extracts (see below), as described in Fig. S12, ESM.

### LC-MS analysis of extracts

Extracts were obtained as described above and re-dissolved in 300 µL of methanol before analysis. 1 µL of methanol solution was separated on C18 Phenomenex columns (100 × 2.1 mm, 1.7 μm) using Dionex Ultimate 3000 HPLC-DAD system coupled to MaXis Impact HD LC-Q-TOF mass-spectrometer (Bruker Daltonics). The LC runtime was 22 min, at a flow rate of 0.6 mL/min. Solvent system: water + 0.1% formic acid (HFo; solvent A), acetonitrile + 0.1% HFo (solvent B); from 95% A to 0% A in 12 min, then 3 min at 0% A, then reversion to 45% A within the remaining 7 min. Ionization was performed in positive mode. Analysis of LC-MS data was performed using the program Compass Data Analysis 4.2 (Bruker Daltonics). See ESM for the access to raw LC-MS data. High-resolution masses were used as a query against Dictionary of Natural Products (version 10.0).

### Purification of limocrocin and derivatives for NMR analysis and bioassays

*S. lividans* 15-H11 pre-culture was grown for 48 h in three 250-mL flasks each containing 50 ml of TSB. 1 ml of the pre-culture was used to inoculate 500-mL flask with glass beads and 100 ml of production medium DNPM. In total we grew 100 flasks of the main culture for 6 days at 28 °C (180 rpm). After separation from glass beads and centrifugation for 10 min at 6,000 rpm 10 L of supernatant was extracted with an equal amount of butanol, evaporated and redissolved in methanol. The obtained extract was separated by size-exclusion chromatography on a Sephadex LH-20 column (Sigma-Aldrich, USA) using methanol as solvent. Semi-preparative chromatography was performed on Dionex Ultimate 3000 UPLC system (Thermo Fisher Scientific, USA) using a 100 mm ACQUITY UPLC BEH C18 1.7 μm column (Waters Corporation, USA). Water with 0.1% formic acid and acetonitrile with 0.1% formic acid were used as the mobile phases.

### NMR spectroscopy

The structure of LIM was determined via multidimensional NMR analysis. ^1^H-NMR, ^13^C-NMR and 2D spectra were recorded at 500 MHz (^1^H) and 126 MHz (^13^C), conducted in the Bruker Avance Neo 500 MHz, equipped with a Prodigy Cryo-probe. LIM was dissolved in dimethyl sulfoxide-d_6_. All 2D experiments were measured using standard experiments from Bruker Topspin software 4.3.0 and non-uniform sampling (NUS). Edited-HSQC provides multiplicity information similar to that of a ^13^C DEPT-135 sequence. Chemical shifts are reported in ppm relative to tetramethylsilane; the solvent was used as the internal standard.

### Bioactivity testing

Limocrocin stock solutions were prepared in dimethyl sulfoxide (DMSO). All microorganisms used in this study were obtained from the German Collection of Microorganisms and Cell Cultures (DMSZ), the American Type Culture Collection (ATCC) or the Coli Genetic Stock Center. Minimum inhibitory concentrations (MICs) were determined using the broth microdilution method according to EUCAST guidelines (ISO 20776-1:2019).

### Cytotoxicity testing

CHO-K1 (chinese hamster ovary cells; ACC 110) were obtained from the German Collection of Microorganisms and Cell Cultures (DSMZ) and cultured under the conditions recommended by the depositor. Cells were propagated in Ham’s F12 medium supplemented with 10% fetal bovine serum (FBS), and seeded at 6 × 10^3^ cells per well of a 96-well plate in 120 µL of complete medium. After 2 h of equilibration (37 °C, 5% CO_2_), the cells were treated with a serial dilution of limocrocins. Limocrocins, doxorubicin as reference, as well as the solvent control (DMSO) were tested as duplicates in two independent experiments. After 5 d of incubation (37 °C, 5% CO_2_), a total of 20 µL of 5 mg/ml MTT (thiazolyl blue tetrazolium bromide) in phosphate-buffered saline (PBS) were added to each well and the cells were further incubated for 2 h at 37 °C before the supernatant was discarded. Subsequently, the cells were washed with 100 µL of PBS and treated with 100 µl of 2-propanol/10 N HCl (250:1) to dissolve formazan granules. Cell viability was measured as a percentage relative to the respective solvent control by measuring the absorbance at 570 nm using a microplate reader (Tecan Infinite M200Pro). GraphPad Prism (version 10.2.3, GraphPad, Boston, MA, USA) was used for sigmoidal curve fitting to determine the IC_50_ values.

### Bioinformatic methods

*S. roseochromogenes* NRRL 3504 genome (NCBI accession number – NZ_CM002285.1) was analyzed using bioinformatic service for in silico BGCs detection - antiSMASH (https://antismash.secondarymetabolites.org/*).* Protein sequences of Lim18 and the top 10 homologues of oxygenases Fma-ABM, CrtP (CruO), and SmcC4 (10 for each of this protein) were retrieved from GenBank (accession numbers are provided in ESM Table S3). Multiple sequence alignment was conducted using the MUSCLE algorithm implemented in MEGA11 Software [40]. A phylogenetic tree was constructed in MEGA11 using the Maximum Likelihood method with 1000 bootstrap replicates to assess the robustness of the inferred topology.

## Electronic supplementary material

Below is the link to the electronic supplementary material.

Supplementary Material 1

## Data Availability

Data is provided within the manuscript and Electronic Supplementary Materials file.

## References

[1] Flatman RH, Eustaquio A, Li SM, Heide L, Maxwell A. Structure-activity relationships of aminocoumarin-type gyrase and topoisomerase IV inhibitors obtained by combinatorial biosynthesis. Antimicrob Agents Chemother. 2006;50:1136–42. 16569821 10.1128/AAC.50.4.1136-1142.2006 PMC1426943

[2] Rückert C, Kalinowski J, Heide L, Apel AK. Draft genome sequence of Streptomyces roseochromogenes subsp. oscitans DS 12.976, producer of the aminocoumarin antibiotic clorobiocin. Genome Announc. 2014;2:e01147–13. 24407645 10.1128/genomeA.01147-13 PMC3886958

[3] Melnyk S, Hrab P, Ostash B. Genomic potential of *Streptomyces roseochromogenes* NRRL 3504 for the production of specialized metabolites: analysis in *silico*. Visnyk Lviv Univ Biol Ser. 2022;87:45–53.

[4] Yan X, Zhang J, Tan H, Liu Z, Jiang K, Tian W, et al. A pair of atypical KAS III homologues with initiation and elongation functions program the polyketide biosynthesis in asukamycin. Angew Chem Int Ed Engl. 2022;61:e202200879. 35218125 10.1002/anie.202200879

[5] Petříčková K, Pospíšil S, Kuzma M, Tylová T, Jágr M, Tomek P, et al. Biosynthesis of colabomycin E, a new manumycin-family metabolite, involves an unusual chain-length factor. ChemBioChem. 2014;15:1334–45. 24838618 10.1002/cbic.201400068

[6] Gorniaková D, Petříček M, Kahoun D, Grabic R, Zelenka T, Chroňáková A, et al. Activation of a cryptic manumycin-type Biosynthetic Gene Cluster of *Saccharothrix espanaensis* DSM44229 by Series of genetic manipulations. Microorganisms. 2021;9:559. 33800500 10.3390/microorganisms9030559 PMC8000086

[7] Rui Z, Petrícková K, Skanta F, Pospísil S, Yang Y, Chen CY, et al. Biochemical and genetic insights into asukamycin biosynthesis. J Biol Chem. 2010;285:24915–24. 20522559 10.1074/jbc.M110.128850 PMC2915727

[8] Brockmann H, Grothe G. Über Actinomycetenfarbstoffe, II. Mitteil.: limocrocin, ein gelber actinomycetenfarbstoff. Chem Ber. 1953;86:1110–5.

[9] Dembitsky VM. Microbiological aspects of unique, rare, and unusual fatty acids derived from natural amides and their pharmacological profile. Microbiol Res. 2022;13:377–417.

[10] Myronovskyi M, Rosenkränzer B, Nadmid S, Pujic P, Normand P, Luzhetskyy A. Generation of a cluster-free *Streptomyces albus* chassis strains for improved heterologous expression of secondary metabolite clusters. Metab Eng. 2018;49:316–24. 30196100 10.1016/j.ymben.2018.09.004

[11] Ahmed Y, Rebets Y, Estévez MR, Zapp J, Myronovskyi M, Luzhetskyy A. Engineering of *Streptomyces lividans* for heterologous expression of secondary metabolite gene clusters. Microb Cell Fact. 2020;19:5. 31918711 10.1186/s12934-020-1277-8 PMC6950998

[12] Siracusa L, Gresta F, Ruberto G. Saffron (Crocus Sativus L.) apocarotenoids: a review of their biomolecular features and biological activity perspectives. Carotenoids: Prop Eff Dis. 2011;6:145–78.

[13] Macdonald G, Norman L, Taylor R. A stille approach to unsaturated amides derived from 2-amino-3-hydroxycyclopentenone: the synthesis of asuka-mABA and limocrocin. Chem Commun. 2010;1996(23):2647–8.

[14] Blin K, Shaw S, Augustijn HE, Reitz ZL, Biermann F, Alanjary M, et al. antiSMASH 7.0: new and improved predictions for detection, regulation, chemical structures and visualisation. Nucleic Acids Res. 2023;51:46–50. 10.1093/nar/gkad344 PMC10320115 37140036

[15] Santos CL, Correia-Neves M, Moradas-Ferreira P, Mendes MV. A walk into the LuxR regulators of Actinobacteria: phylogenomic distribution and functional diversity. PLoS ONE. 2012;7(10):e46758. 23056438 10.1371/journal.pone.0046758 PMC3466318

[16] Lin HC, Tsunematsu Y, Dhingra S, Xu W, Fukutomi M, Chooi YH, et al. Generation of complexity in fungal terpene biosynthesis: discovery of a multifunctional cytochrome P450 in the fumagillin pathway. J Am Chem Soc. 2014;136:4426–36. 24568283 10.1021/ja500881e PMC3985917

[17] Zhang W, Bolla ML, Kahne D, Walsh CT. A three enzyme pathway for 2-amino-3-hydroxycyclopent-2-enone formation and incorporation in natural product biosynthesis. J Am Chem Soc. 2010;132:6402–11. 20394362 10.1021/ja1002845 PMC2866186

[18] Hanajima S, Ishimaru K, Sakano K, Roy SK, Inouye Y, Nakamura S. Inhibition of reverse transcriptase by limocrocin. J Antibiot (Tokyo). 1985;38:803–5. 2410398 10.7164/antibiotics.38.803

[19] Lasch C, Gummerlich N, Myronovskyi M, Palusczak A, Zapp J, et al. Loseolamycins: A Group of New Bioactive Alkylresorcinols Produced after Heterologous expression of a type III PKS from *Micromonospora endolithica*. Molecules. 2020;25:4594. 33050154 10.3390/molecules25204594 PMC7587189

[20] Besl H, Bresinsky A, Meixner B, Mocek U, Steglich W. Verpacrocin, ein Polyenpigment aus Mycelkulturen von *Verpa digitaliformis* (Pers.) Fr. (Ascomycetes). Zeitschrift für Naturforschung C. 1983;38,492–493.

[21] Kahner L, Dasenbrock J, Spiteller P, Steglich W, Marumoto R, Spiteller M. Polyene pigments from fruit-bodies of *Boletus laetissimus* and *B. Rufo-Aureus* (basidiomycetes). Phytochemistry. 1998;49:1693–7. 11711083 10.1016/s0031-9422(98)00319-7

[22] Gruber G, Steglich W. Calostomal, a Polyene pigment from the Gasteromycete *Calostoma cinnabarinum* (Boletales). Z für Naturforschung. 2007;62:129–31.

[23] Erdtman H. Corticrocin, a mycorrhiza pigment. Nature. 1947;160(4062):331. 20261771 10.1038/160331a0

[24] Löhr NA, Eisen F, Thiele W, Platz L, Motter J, Hüttel W. Unprecedented mushroom polyketide synthases produce the Universal Anthraquinone Precursor. Angew Chem Int Ed Engl. 2022;61:e202116142. 35218274 10.1002/anie.202116142 PMC9325552

[25] Havaux M. Carotenoid oxidation products as stress signals in plants. Plant J. 2014;79:597–606. 24267746 10.1111/tpj.12386

[26] Getino M, Fernández-López R, Palencia-Gándara C, Campos-Gómez J, Sánchez-López JM, Martínez M. Tanzawaic acids, a chemically novel set of bacterial conjugation inhibitors. PLoS ONE. 2016;11:e0148098. 26812051 10.1371/journal.pone.0148098 PMC4727781

[27] Cabezón E, de la Cruz F, Arechaga I. Conjugation inhibitors and their potential use to prevent dissemination of Antibiotic Resistance genes in Bacteria. Front Microbiol. 2017;8:2329. 29255449 10.3389/fmicb.2017.02329 PMC5723004

[28] Bilyk O, Brötz E, Tokovenko B, Bechthold A, Paululat T, Luzhetskyy A. New simocyclinones: surprising evolutionary and biosynthetic insights. ACS Chem Biol. 2016;11:241–50. 26566170 10.1021/acschembio.5b00669

[29] Kieser T, Bibb MJ, Buttner MJ, Chater KF, Hopwood DA. Practical Streptomyces genetics. Norwich: John Innes Foundation; 2000.

[30] Rodríguez Estévez M, Myronovskyi M, Gummerlich N, Nadmid S, Luzhetskyy A. Heterologous expression of the Nybomycin Gene Cluster from the Marine strain *Streptomyces albus* subsp. *Chlorinus* NRRL B-24108. Mar Drugs. 2018;16:435. 30400361 10.3390/md16110435 PMC6265801

[31] Green MR, Sambrook J, Molecular Cloning. A Laboratory Manual. 4th ed. Plainview, NY, USA: Cold Spring Harbor Laboratory Press; 2012.

[32] Sambrook J, Russell DW. Molecular cloning: a Laboratory Manual. 3rd ed. NY, USA: Cold Spring Harbor Laboratory Press; 2001.

[33] Gust B, Challis GL, Fowler K, Kieser T, Chater KF. PCR-targeted *Streptomyces* gene replacement identifies a protein domain needed for biosynthesis of the sesquiterpene soil odor geosmin. Proc Natl Acad Sci U S A. 2003;100:1541–6. 12563033 10.1073/pnas.0337542100 PMC149868

[34] Horbal L, Luzhetskyy A. Dual control system - A novel scaffolding architecture of an inducible regulatory device for the precise regulation of gene expression. Metab Eng. 2016;37:11–23. 27040671 10.1016/j.ymben.2016.03.008 PMC4915818

[35] Huang J, Shi J, Molle V, Sohlberg B, Weaver D, Bibb M, et al. Cross-regulation among disparate antibiotic biosynthetic pathways of *Streptomyces coelicolor*. Mol Microbiol. 2005;58:1276–87. 16313616 10.1111/j.1365-2958.2005.04879.x

[36] Herrmann S, Siegl T, Luzhetska M, Jilg LP, Welle E, Erb A, et al. Site-specific recombination strategies for engineering actinomycete genomes. Appl Environ Microbiol. 2012;78:1804–12. 22247163 10.1128/AEM.06054-11 PMC3298146

[37] Fu J, Wenzel SC, Perlova O, Wang J, Gross F, Tang Z, et al. Efficient transfer of two large secondary metabolite pathway gene clusters into heterologous hosts by transposition. Nucleic Acids Res. 2008;36:e113. 18701643 10.1093/nar/gkn499 PMC2553598

[38] Blodgett J, Thomas P, Li G, Velasquez J, van der Donk W, Kelleher NL, et al. Unusual transformations in the biosynthesis of the antibiotic phosphinothricin tripeptide. Nat Chem Biol. 2007;3:480–5. 17632514 10.1038/nchembio.2007.9 PMC4313788

[39] Bilyk B, Luzhetskyy A. Unusual site-specific DNA integration into the highly active pseudo-*attB* of the *Streptomyces albus* J1074 genome. Appl Microbiol Biotechnol. 2014;98:5095–104. 24566921 10.1007/s00253-014-5605-y

[40] Tamura K, Stecher G, Kumar S. MEGA11: Molecular Evolutionary Genetics Analysis Version 11. Mol Biol Evol. 2021;38:3022–7. 33892491 10.1093/molbev/msab120 PMC8233496

