# Supplementary material for: Uncovering the genetic basis of antiviral polyketide limocrocin biosynthesis through heterologous expression
Source: Microb Cell Fact. 2025 Jan 13;24:17. doi: 10.1186/s12934-024-02621-9 (PMC11727611; doi:10.1186/s12934-024-02621-9)
Supplement: Supplementary file 1 — Supplementary Material 1 [file 12934_2024_2621_MOESM1_ESM.pdf]

Electronic Supplementary Materials for

Uncovering the genetic basis of antiviral polyketide limocrocin biosynthesis through heterologous expression

Sofiia Melnyk[1,2], Marc Stierhof[3], Dmytro Bratiichuk[3,4], Franziska Fries[4,5], Rolf Müller[4,5,6],Yuriy Rebets[2,6], Andriy Luzhetskyy[3,6,*], Bohdan Ostash[1,6,*]

[1]Department of Genetics and Biotechnology, Ivan Franko National University of Lviv, Hrushevskoho st. 4, Lviv 79005, Ukraine
[2]Explogen LLC, Volodymyra Velykoho st. 16, Lviv 79032, Ukraine
[3]Helmholtz Institute for Pharmaceutical Research Saarland (HIPS), UdS Campus, Bld. E8.1, 66123 Saarbrücken, Germany.
[4]Helmholtz Institute for Pharmaceutical Research Saarland (HIPS), Helmholtz Centre for Infection Research (HZI), Saarland University, 66123 Saarbrücken, Germany.
[5]German Center for Infection Research (DZIF), Partner Site Hannover-Braunschweig, 38124 Braunschweig, Germany.
[6]German-Ukrainian Core of Excellence in Natural Products Research (CENtR), Zelena st. 20, Lviv 79005, Ukraine

*Corresponding authors:
Biology:
Prof. B. Ostash
Ivan Franko National University of Lviv
Department of Genetics and Biotechnology,
Hrushevskoho st. 4, Rm. 102,
Lviv 79005
Tel.: +38 067 397 30 36 e-mail: bohdan.ostash@lnu.edu.ua
Chemistry:
Prof. A. Luzhetskyy
Saarland University, HIPS
UdS Campus Bldg C2.3
Saarbruecken 66123
Tel.: +49 681 302-70215 e-mail: Andriy.Luzhetskyy@helmholtz-hips.de

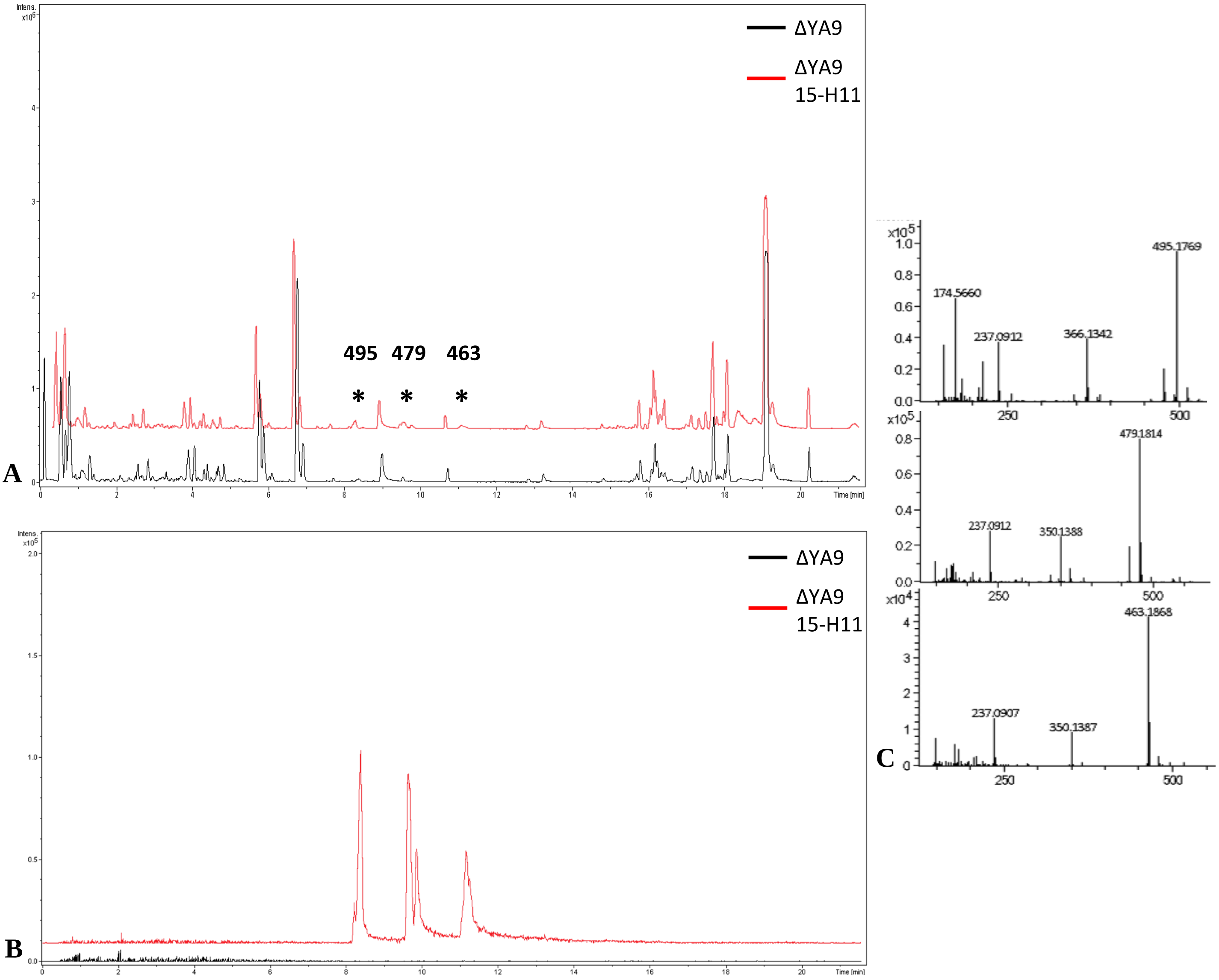

Fig. S1. (**A**) The base peak chromatogram of ΔYA9 (black) and ΔYA9 carrying 15-H11 cosmid (red) extracts ($[M+H]^+$). (**B**) Extracted mass peaks of LIM, *m/z* 463, and its derivatives *m/z* 479 and *m/z* 495 ($[M+H]^+$). (**C**) Mass spectra associated with peaks having retention time 8.4, 9.6, and 11.2 min from the chromatograms to the left (*m/z*).

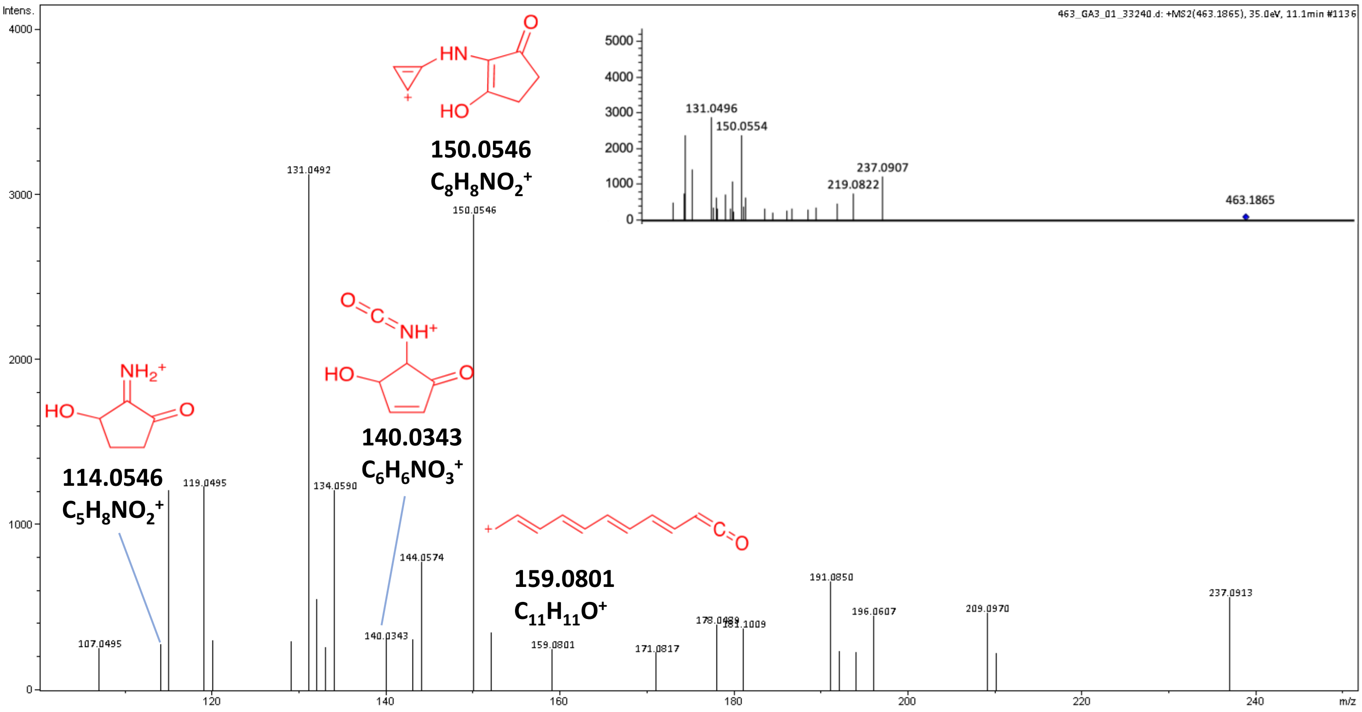

Fig. S2. Collision-induced dissociation spectra of compound with *m/z* 463 (MS/MS in positive ionization mode, upper right inset). The structure of the fragments was predicted by CFM-ID 4.0 / Mass Frontier 8.0 Software (Thermo Scientific).

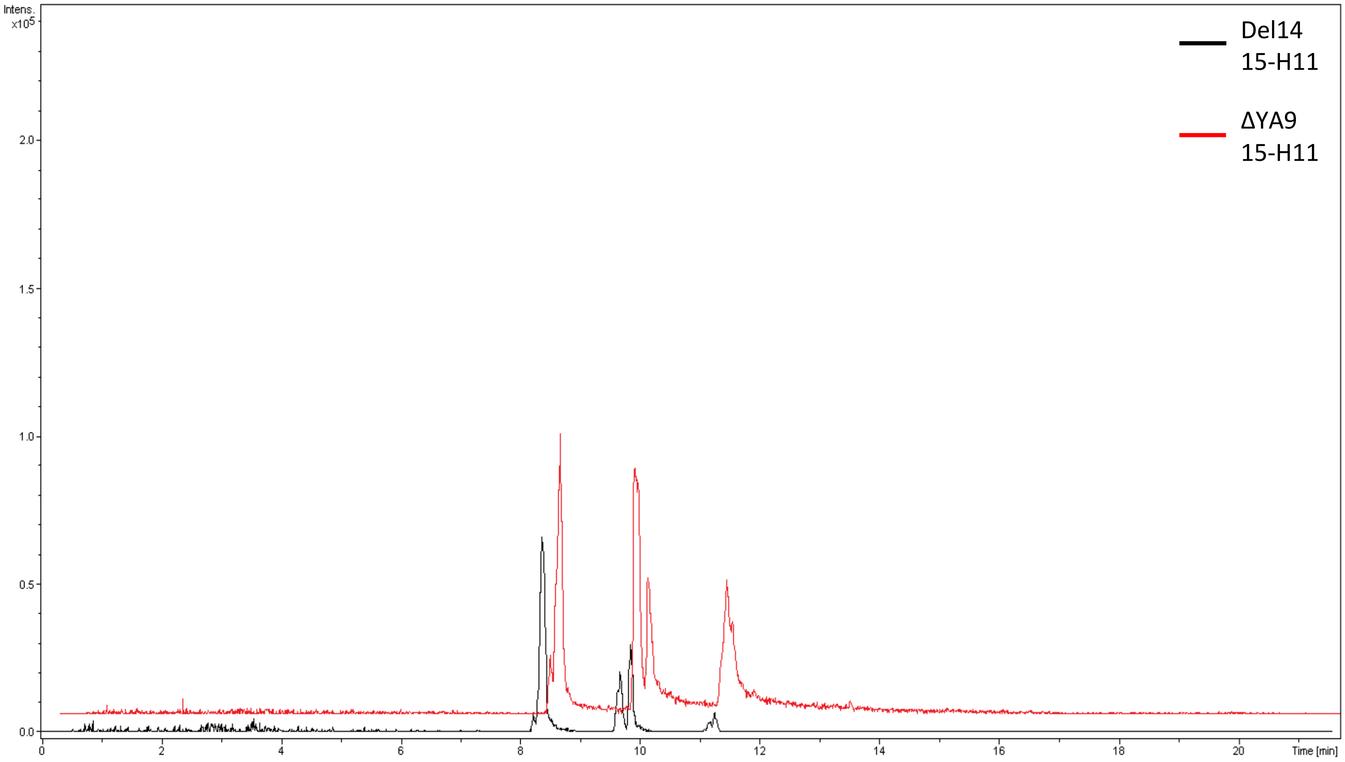

Fig. S3. Comparison of limocrocins production by heterologous hosts Del14 (black) and ΔYA9 (red) ($[M+H]^+$). The three mass peaks correspond to compounds with *m/z* 495, 479 and 463. Trace chromatograms represent typical result of four independent experiments.

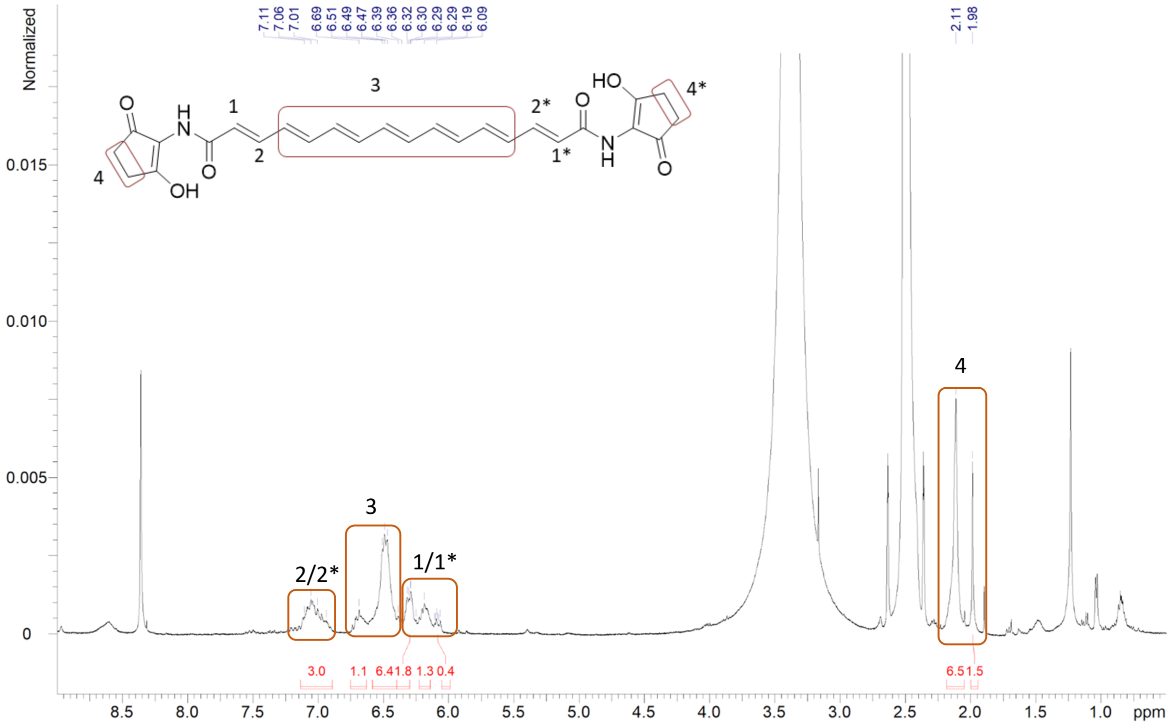

Fig. S4. ^{1}H-NMR spectrum (500 MHz, DMSO-d6) of isomerically impure limocrocin. Related signals are labeled with red boxes and numbers. The combined integral values from the olifinic CH groups (1/1*, 2/2*, 3) and the combined integral values of 4 are in 14/8 ratio.

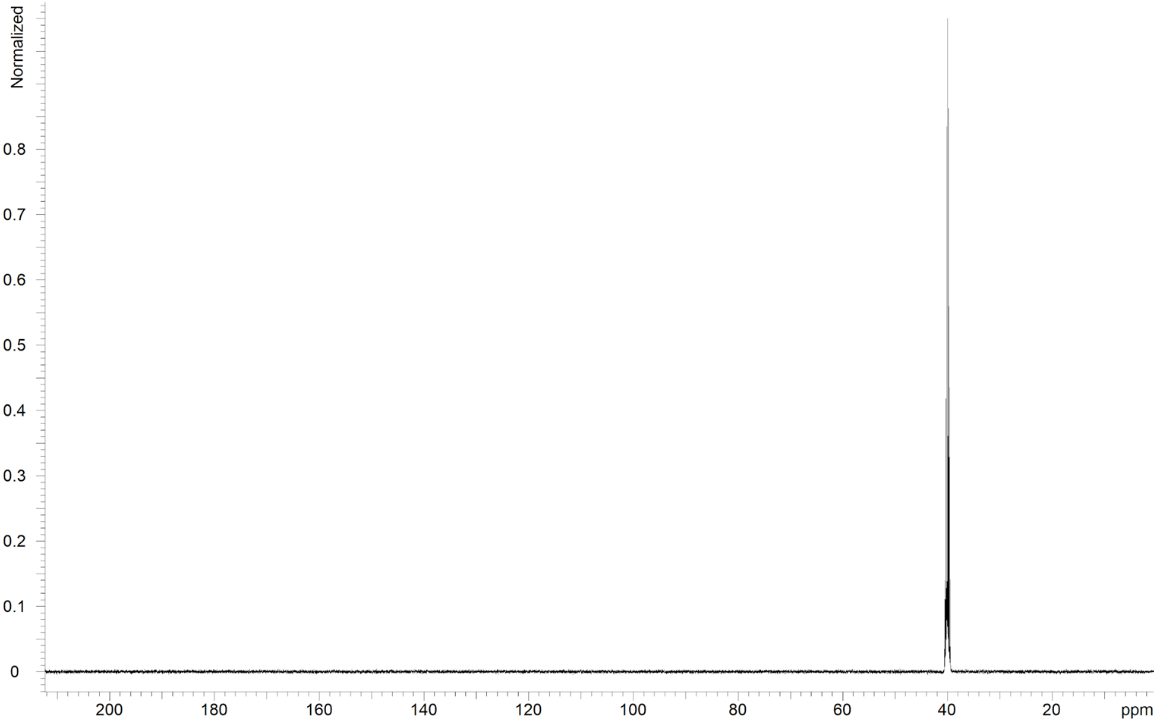

Fig. S5. ^{13}C-NMR spectrum (125 MHz, DMSO-d6) of isomerically impure limocrocin.

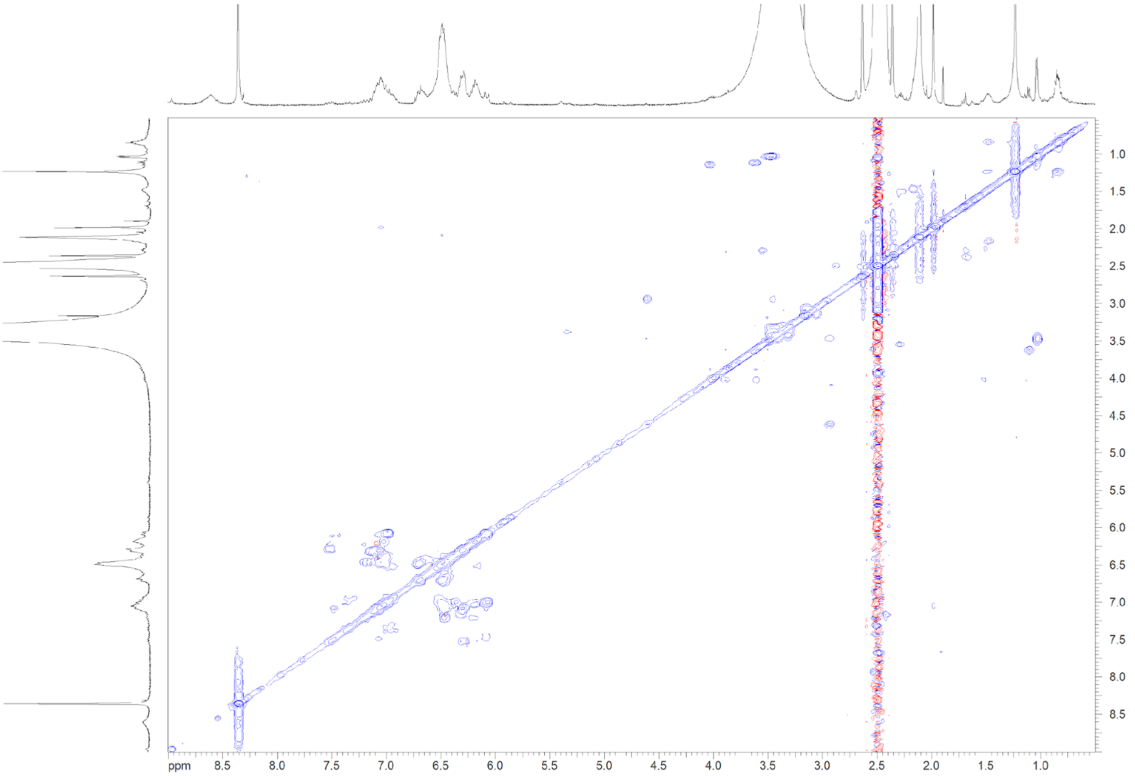

Fig. S6. COSY spectrum (500 MHz, 50% NUS, DMSO-d6) of isomerically impure limocrocin.

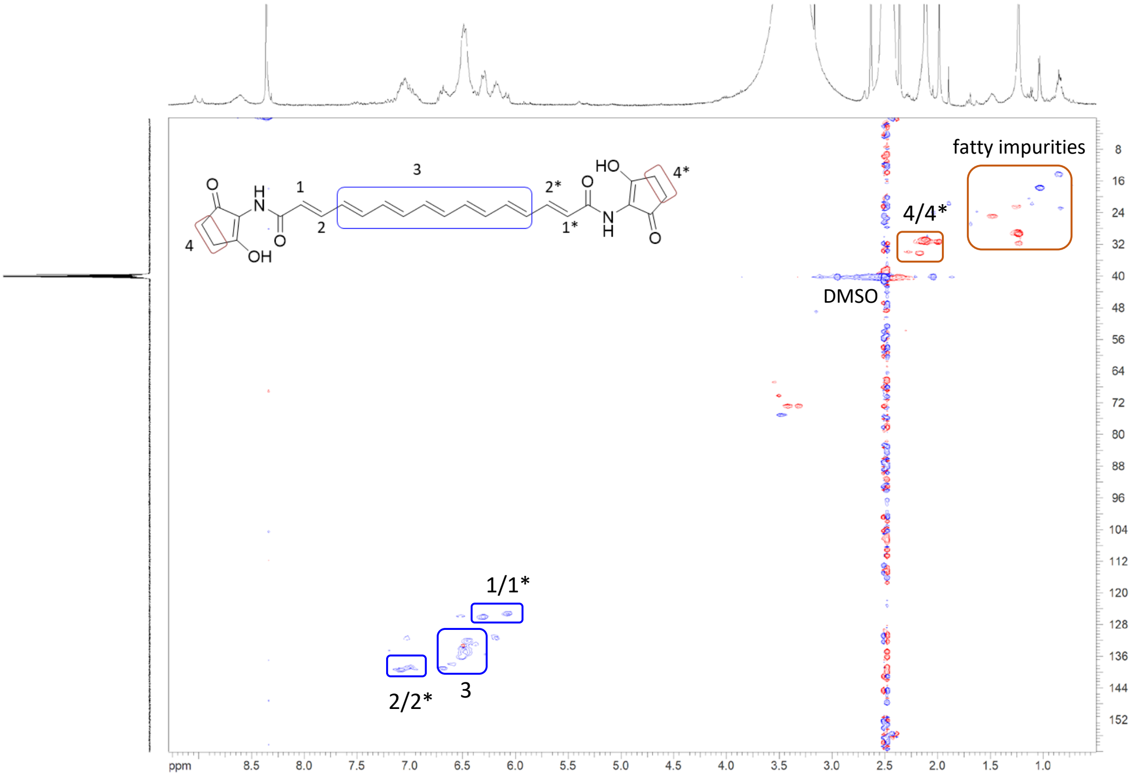

Fig. S7. Edited-HSQC spectrum (500 MHz, 50% NUS, DMSO-d6) of isomerically impure limocrocin. Related signals are labeled with blue/red boxes and numbers.

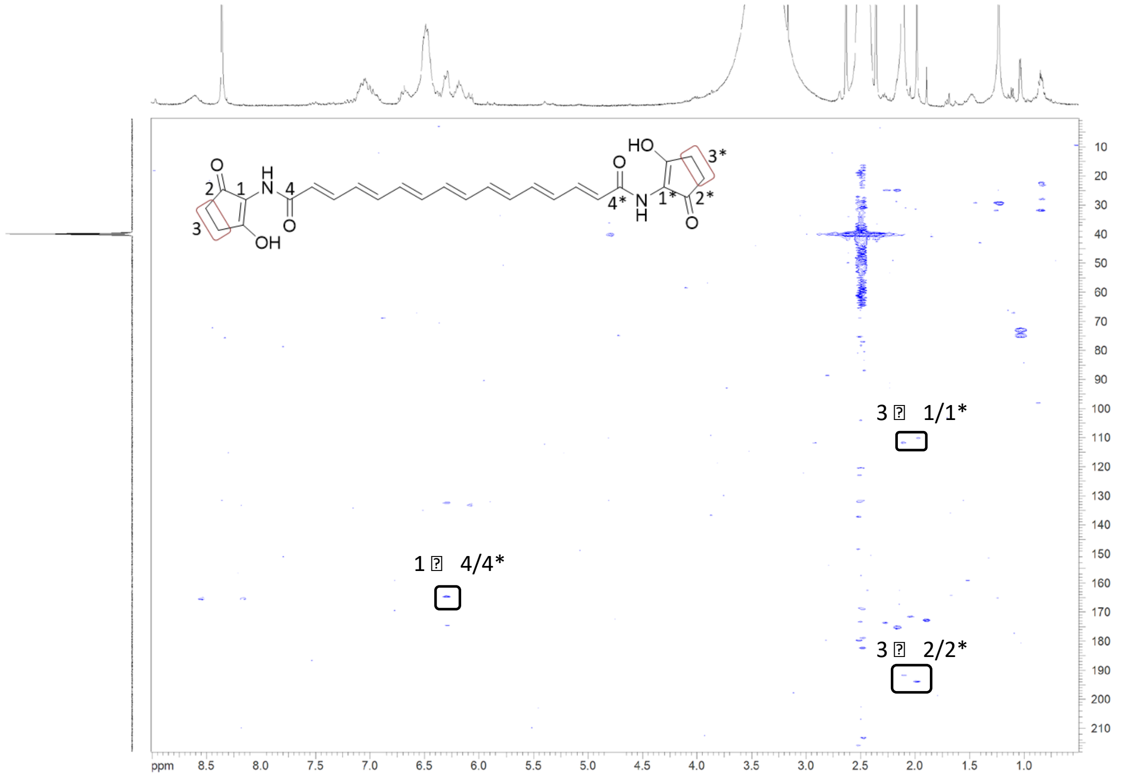

Fig. S8. HMBC spectrum (500 MHz, 50% NUS, DMSO-d6) of isomerically impure limocrocin. Observable key HMBC correleations are labeled with black boxes and numbers.

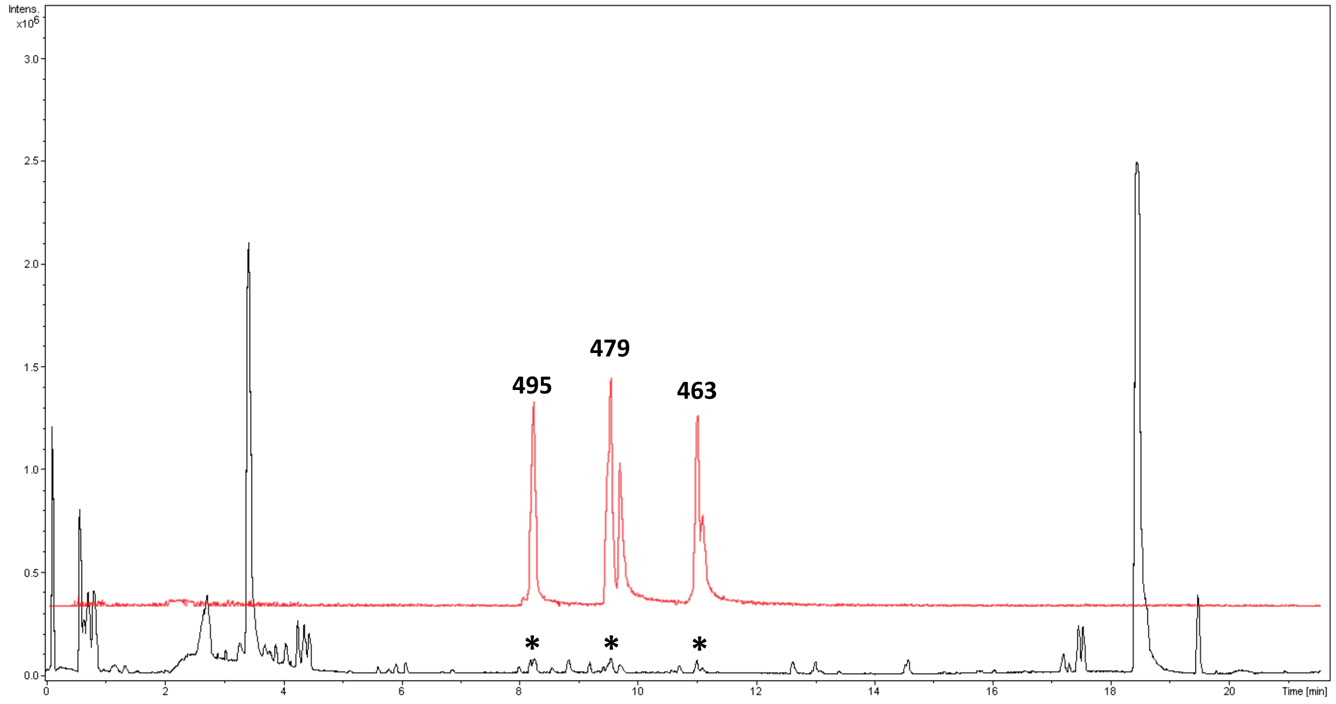

Fig. S9. Limocrocins detected in NRRL 3504 crude extract. Black trace - the base peak chromatogram, red trace - chromatogram with extracted peaks of compounds with *m/z* 463, 479 and 495 (MS data in positive ionization mode).

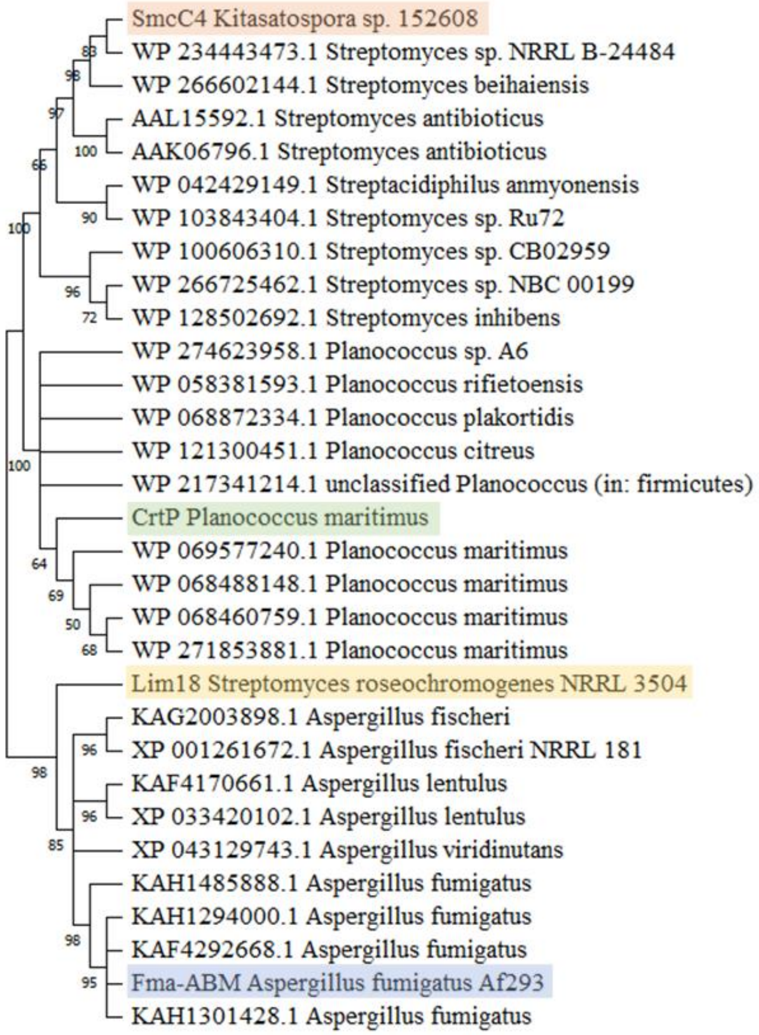

Fig. S10. Full ML tree of Lim18 protein and its functional counterparts from various specialized metabolic pathways (shown in a collapsed form in the main Fig. 5). Access numbers to all protein sequences used to build this tree are given above in the ESM Table S1. Colored background marks proteins with experimentally verified functions (except Lim18). SmcC4 and CrtP fall into family of carotenoid oxygenases, Fma-ABM functions as monooxygenase.

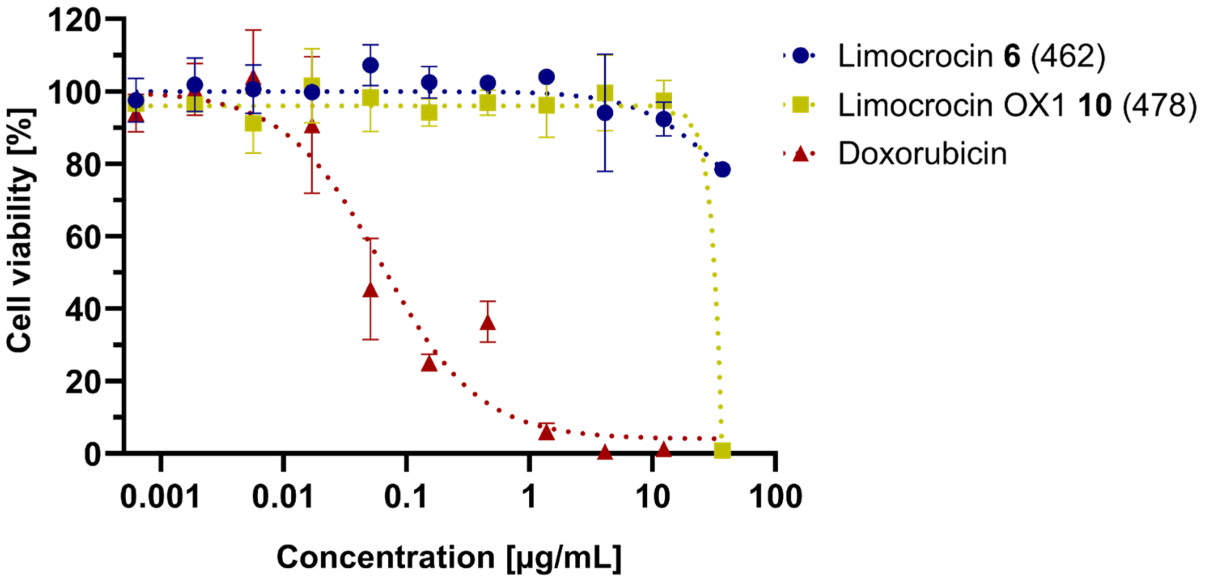

Fig. S11. The curves used for IC_{50} determination against the CHO cells.

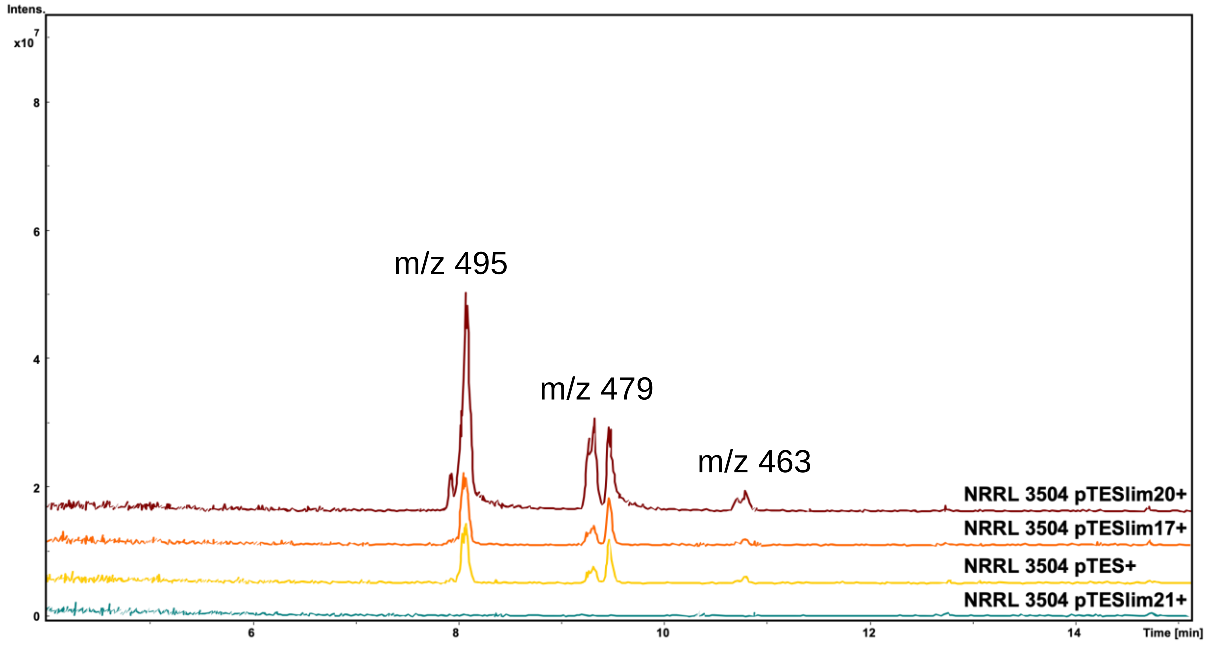

Fig. S12. Overlaid EIC traces for mass-peaks of 495, 479 and 463 Da ($[M+H]^+$) found in the extracts from *S. roseochromogenes* NRRL3504 strains expressing pTESlim20, pTESlim17, pTESlim21 and empty vector pTES. LIM-related mass peaks are marked with cation mass. The extracts were prepared from equal amounts of the biomass, as described in the main text. The chromatogram represents typical result of three independent experiments.

Table S1. GenBank accession numbers and % sequence identity of proteins from BGCs highly syntenic to *lim* gene cluster

**Protein encoded by *lim* BGC**	**Homologue from *Kitasatospora* sp. RG8 BGC / % ID**	**Homologue from *Streptomyces* sp. CB01881 BGC / % ID**	**Homologue from colabomycin BGC of *Streptomyces aureus* / % ID**
Lim1			
Lim2	WP_245204864.1 / 59%	WP_148641358.1 / 59%	AIL50189.1 (ColD3) / 58%
Lim3	WP_209412476.1 / 75%	WP_148641359.1 / 75%	AIL50190.1 (ColD2) / 67%
Lim4			
Lim5	WP_209412477.1 / 81%	WP_148641360.1 / 80%	AIL50191.1 (ColD1) / 54%
Lim6	WP_209412476.1 / 84%	WP_148641359.1 / 84%	AIL50190.1 (ColD2) / 73%
Lim7	WP_209412618.1 / 86%	WP_148641357.1 / 86%	AIL50168.1 (ColC10) / 56%
Lim8	WP_209412474.1 / 68%	WP_148641356.1 / 67%	
Lim9	WP_256463504.1 / 82%	WP_162002412.1 / 82%	
Lim10	WP_148641354.1 / 87%	WP_148641354.1 / 87%	AIL50167.1 (ColC5) / 61%
Lim11	WP_209412472.1 / 92%	WP_148641353.1 / 91%	AIL50166.1 (ColC4) / 55%
Lim12	WP_209412471.1 / 86%	WP_148641352.1 / 87%	AIL50165.1 (ColC3) / 50%
Lim13	WP_245204862.1 / 77%	WP_148641351.1 / 76%	
Lim14	WP_256463503.1 / 87%	WP_206670899.1 / 89%	AIL50164.1 (ColC16) / 53%
Lim15	WP_209412468.1 / 67%	WP_153882756.1 / 67%	
Lim16	WP_245204827.1 / 89%	WP_148641350.1 / 85%	
Lim17	WP_245204860.1 / 75%		AIL50186.1 (ColR1) / 52%
Lim18	WP_209412466.1 / 88%		
Lim19	WP_209412465.1 / 85%		
Lim20	WP_209412464.1 / 68%		
Lim21			

Table S2. Oligonucleotide primers, used in this work

Name	Sequence (5′-3′)*	Purpose
cos15AChF	TTATGGATCTCCATCGACTAAACGTC	Sequencing of *lim*
cos15AChR	CCACAGTTAACTGCGGTCAAGATAT	BGC
lim_gap_F	AGTTTCGACGCCTCCGGAT	Sequencing of gap in
lim_gap_R	TCTCGTAGGCGTCCTCATGG	*lim* BGC
Δlim3hyg_F	AATGAACCTGCATCTGGAGACTGACA	*lim3* deletion
Δlim3hyg_R	CAAGAATCCCTGTTAC	
	GGTCATGCACCATGCCGGTCGAGGCG	
	CCGGGGGCGGTGTCC	
Δlim3_ChF	GATGATTCCTACGCGAGCCTG	Confirming of *lim3*
Δlim3_ChR	TGGTGCCAGAAGTTGCCGAC	deletion
lim3_XbaI_F	AAATCTAGACACGGTCATGCACCA	Complementation of
lim3_EcoRI_R	AAAGAATTCGAACGAAATGAACCTGC	*lim3* deletion
	A	
Δlim6hyg_F	AGACGAACGGAATGAACCGACTGACA	*lim6* deletion
Δlim6hyg_R	CAAGAATCCCTGTTAC	
	TCTCTTTCAGCTCTGCTGAGGCGCCGG	
	GGGCGGTGTCC	
Δlim6_ChF	ATGATTCCTACGCGAGATGGC	Confirming of *lim6*
Δlim6_ChR	TCACCGAGCTTTTCCTCGGTG	deletion
lim17_EcoRV_F	AAAGATATCGAAGTGAGGCACCG	*lim17* cloning into
lim17_EcoRI_R	TTTGAATTCGTCGATGCTCCTTCG	expression vector
lim20_KpnI_F	AAAGGTACCGGCCGGTATAGTTT	*lim20* cloning into
lim20_BglII_R	TTTAGATCTGGTCGACCGAGGA	expression vector
lim21_EcoRV_F	AAAGATATCATACTAGCCGAGGAGG	*lim21* cloning into
lim21_EcoRI_R	TTTGAATTCTTCGTGCATATAAGGGGG	expression vector

***** restriction endonuclease recognition sites are underlined.

Table S3. NCBI accession numbers of proteins and corresponding species names used in the phylogenetic reconstruction of Lim18 homologs

Protein	Accession	Organism
Lim18	WP_023548514.1	*Streptomyces roseochromogenes* NRRL 3504
CrtP	BDQ10374.1	*Planococcus maritimus*
	WP_068488148.1	*Planococcus maritimus*
	WP_068460759.1	*Planococcus maritimus*
	WP_069577240.1	*Planococcus maritimus*
	WP_271853881.1	*Planococcus maritimus*
	WP_217341214.1	unclassified *Planococcus* (in: firmicutes)
	WP_058381593.1	*Planococcus rifietoensis*
	WP_068872334.1	*Planococcus plakortidis*
	WP_274623958.1	*Planococcus sp.* A6
	WP_121300451.1	*Planococcus citreus*
Fma-ABM	Q4WAZ2.1	*Aspergillus fumigatus* Af293
	KAH1294000.1	*Aspergillus fumigatus*
	KAF4292668.1	*Aspergillus fumigatus*
	KAH1301428.1	*Aspergillus fumigatus*
	KAH1485888.1	*Aspergillus fumigatus*
	KAG2003898.1	*Aspergillus fischeri*
	XP_001261672.1	*Aspergillus fischeri* NRRL 181
	XP_043129743.1	*Aspergillus viridinutans*
	KAF4170661.1	*Aspergillus lentulus*
	XP_033420102.1	*Aspergillus lentulus*
SmcC4	ALT05957.1	*Kitasatospora* sp. 152608
	WP_234443473.1	*Streptomyces* sp. NRRL B-24484
	WP_266602144.1	*Streptomyces beihaiensis*
	AAL15592.1	*Streptomyces antibioticus*
	AAK06796.1	*Streptomyces antibioticus*
	WP_042429149.1	*Streptacidiphilus anmyonensis*
	WP_103843404.1	*Streptomyces* sp. Ru72
	WP_100606310.1	*Streptomyces* sp. CB02959
	WP_266725462.1	*Streptomyces* sp. NBC_00199
	WP_128502692.1	*Streptomyces inhibens*
